# Targeting polyunsaturated fatty acids desaturase FADS1 inhibits renal cancer growth via ATF3-mediated ER stress response

**DOI:** 10.1016/j.biopha.2025.118006

**Published:** 2025-03-22

**Authors:** Gioia Heravi, Zhenjie Liu, Mackenzie Herroon, Alexis Wilson, Yang-Yi Fan, Yang Jiang, Nivisa Vakeesan, Li Tao, Zheyun Peng, Kezhong Zhang, Jing Li, Robert S. Chapkin, Izabela Podgorski, Wanqing Liu

**Affiliations:** a Department of Pharmaceutical Sciences, Eugene Applebaum College of Pharmacy and Health Sciences, Wayne State University, Detroit, MI 48201, USA; b Department of Pharmacology, School of Medicine, Wayne State University, Detroit, MI 48201, USA; c Department of Nutrition, Program in Integrative Nutrition and Complex Diseases, Texas A&M University, College Station, TX 77843, USA; d Department of Physiology, Wayne State University School of Medicine, Detroit, MI 48201, USA; e Center for Molecular Medicine and Genetics, Wayne State University, Detroit, MI 48201, USA; f Department of Biochemistry, Microbiology, and Immunology, School of Medicine, Wayne State University, Detroit, MI 48201, USA; g Department of Oncology, School of Medicine, Wayne State University, and Karmanos Cancer Institute, Detroit, MI 48201, USA; h CPRIT Regional Center of Excellence in Cancer Research, Texas A&M University, College Station, TX 77843, USA

**Keywords:** FADS1, PUFA, Kidney cancer, ER stress, ATF3

## Abstract

**Objective:**

Fatty Acid Desaturase 1 (FADS1) is a rate-limiting enzyme controlling the bioproduction of long-chain polyunsaturated fatty acids (PUFAs). Increasing studies suggest that FADS1 is a potential cancer target. Our previous research has demonstrated the significant role of FADS1 in cancer biology and patient survival, especially in kidney cancers. We aim to explore the underlying mechanism in this study.

**Method and results:**

We found that pharmacological inhibition or knockdown of the expression of FADS1 significantly reduced the intracellular conversion of long-chain PUFAs, effectively inhibits renal cancer cell proliferation, and induces cell cycle arrest. The stable knockdown of FADS1 also significantly inhibits tumor formation *in vivo*. Mechanistically, we showed that while FADS1 inhibition induces endoplasmic reticulum (ER) stress, FADS1 expression is augmented by ER-stress inducer, suggesting a necessary role of PUFA production in response to ER stress. FADS1-inhibition sensitized cellular response to ER stress inducers, leading to cell apoptosis. Also, FADS1 inhibition-induced ER stress leads to activation of the PERK/eIF2α/ATF4/ATF3 pathway. Inhibiting PERK or knockdown of ATF3 rescued FADS1 inhibition-induced ER stress and cell growth suppression, while ATF3-overexpression aggravates the FADS1 inhibition-induced cell growth suppression and leads to cell death. Metabolomic analysis revealed that FADS1 inhibition results in decreased level of UPD-N-Acetylglucosamine, a critical mediator of the unfolded protein response, as well as impaired biosynthesis of nucleotides, possibly accounting for the cell cycle arrest.

**Conclusion:**

Our findings suggest that PUFA desaturation is crucial for rescuing cancer cells from persistent ER stress, supporting FADS1 as a new therapeutic target.

## 1. Introduction

Metabolic reprogramming, a hallmark of most types of cancers, plays a vital role in cancer cell growth, energy metabolism, storage, and signaling [1–3]. Targeting genes or enzymes involved in metabolism is a promising strategy to inhibit cancer cell growth [4,5]. Previous studies have demonstrated that fatty acid metabolism, especially the desaturation process, is critical for cancer progression and therapeutic evasion [6,7]. This is exemplified by the role of Stearoyl-CoA Desaturase (SCD), the rate-limiting enzyme that catalyzes the conversion of saturated fatty acids (SFAs) into monounsaturated fatty acids (MUFAs) [8,9]. However, whether the biosynthesis system for polyunsaturated fatty acids (PUFAs) also plays an important role in cancer metabolism and cancer survival remain less characterized.

PUFAs, especially long chain (LC)-PUFAs such as typical n-6 PUFA arachidonic acid (AA), and n-3 PUFAs eicosatetraenoic acid (EPA), and docosahexaenoic acid (DHA), are important signaling molecules and key components of biomembrane phospholipids [10,11]. PUFAs and their derivatives have been implicated to play broad functions in cancer impacting various stages from tumorigenesis to cancer progression and metastasis [12–16]. Previous research has shown that Fatty Acid Desaturase 1 (FADS1), a rate-limiting enzyme directly controlling the bioproduction of AA and EPA, plays a significant role in the growth of various cancer cells [7]. FADS1 was observed to be overexpressed in colon, pancreas, breast, and laryngeal cancers [17]. In our previous study, we have systematically investigated the role of FADS1 expression in all cancers of The Cancer Genome Atlas Program (TCGA) datasets and demonstrated broad associations between FADS1 expression and cancer aggressiveness, tumor microenvironment changes, as well as cancer patient survival, with the most significant correlations identified in kidney cancers [7].

Clear cell renal cell carcinoma (ccRCC) is the most common subtype of kidney cancer. The 5-year survival rate for ccRCC is 50–69 %, and for distant stages the survival rate drops to 10 % [18]. Treatment for localized RCC involves surgery [19], but about 30 % of patients eventually develop metastatic RCC (mRCC), which is resistant to conventional chemotherapy [20]. The current first-line treatment for mRCC is a combination of immunotherapy and anti-angiogenic tyrosine kinase inhibitors targeting vascular endothelial growth factor (VEGF) [21–23]. However, these targeted therapies are known to have limitations and there is a need for identifying new targets and drugs to treat these cancers.

In this study, we investigated the role and detailed molecular mechanism underlying FADS1 on controlling renal cancer cell proliferation and tumor growth. Our findings demonstrated that inhibiting FADS1 activity or knockdown of FADS1 expression can impede renal cancer cell proliferation and induce cell cycle arrest. Our *in vivo* mouse tumorigenesis experiments further indicate that limiting FADS1 expression reduces tumor formation and growth. Mechanistically, we demonstrated that FADS1 plays a reciprocal role in ER stress and downregulation results in inhibition of tumor growth through induction of PERK/eIF2α/ATF4/ATF3-mediated ER stress response. Collectively, our findings suggest that FADS1 could be a novel therapeutic target for renal cancer carcinoma.

## 2. Methods

### 2.1. Cell culture

The human renal cancer cell lines (786-o, A498, ACHN, Caki-1), RPTEC, and HEK 293 were purchased from the American Type Culture Collection (ATCC, VA, USA). 786-o, A498, ACHN, and HEK 293 were maintained in Dulbecco’s modified Eagle’s medium (DMEM, Sigma-Aldrich, St Louis, MO, USA) supplemented with 10 % fetal bovine serum (FBS, Thermo Fisher Scientific, Waltham, MA, USA) and 1 % penicillin-streptomycin solution (Life Technologies, Carlsbad, CA, USA). Caki-1 was maintained in McCoy’s 5 A medium (Life Technologies, Carlsbad, CA, USA) with 10 % FBS and 1 % penicillin-streptomycin solution. RPTEC was maintained in basal medium (ATCC, VA, USA) consisting of renal epithelial cell growth kit (ATCC, VA, USA) and 1 % penicillin-streptomycin solution. All cells were cultured in a 5 % CO_2_ 37 °C humidified incubator.

### 2.2. Transduction

786-o and A498 cells were seeded in 12-well plates (Fisher Scientific, Hampton, NH, USA) and transduced with virus particles when reaching 70 % confluency. Multiplicity of infection (MOI) of 1 × 10^5^ of specific shRNA lentivirus particles (sh-scramble, sh-FADS1, or sh-ATF3; Santa Cruz Biotechnology, Dallas, TX, USA; detailed sequence information of each shRNA was shown in the Supplemental Table 1) or gene overexpression lentivirus particles (OE-stuffer and OE-ATF3; VectorBuilder Inc, Chicago, IL, USA; detail sequence information of each was shown in the Supplemental Table 1) were added into the culture medium with 5 μg/mL polybrene (Santa Cruz Biotechnology, Dallas, TX, USA). Transfection was discontinued by changing the culture medium 24 h after treatment. Cells were treated with 10 μg/mL puromycin dihydrochloride (for gene knockdown assay, Santa Cruz Biotechnology, Dallas, TX, USA) or 10 μg/mL hygromycin (for gene overexpression assay, Thermo Fisher, Waltham, MA, USA) for 24 h to elute the non-transfected cells. The condition of the gene and protein knockdown were evaluated by qPCR assay and Western blot assay which is shown in the “RNA extraction and RT-qPCR” and “Western blot” section.

### 2.3. FADS1 inhibition assay

To investigate the impact of FADS1 in cancer cell growth, equal number of renal cancer cells, including the transfected 786-o and A498 cells, were cultured with increasing concentrations (20, 200, 2000 nM; Dimethyl sulfoxide [DMSO] as vehicle) of D5D-IN-326 (FADS1 inhibitor; Sigma-Aldrich, St Louis, MO, USA) for 48 and 96 h. The total cell number and condition of the cells were counted with hemocytometer or evaluated by the immunofluorescence assay which was showed in the “Immunofluorescence staining” section.

### 2.4. PERK inhibition assay

To investigate the mechanism of FADS1 triggered renal cancer cell growth restriction, equal number of renal cancer cells, including the transfected 786-o and A498 cells, were cultured with 2000 nM D5D-IN-326 (FADS1 inhibitor; Sigma-Aldrich, St Louis, MO, USA) and 50 nM PERK inhibitor (GSK 2606414, R&D systems, Minneapolis, MN, USA) for 48 and 96 h (DMSO as vehicle for control groups). The total cell number and condition of the cells were evaluated by the immunofluorescence assay which was showed in the “Immunofluorescence staining” section.

### 2.5. ER stress stimulation assay

To investigate the role of ER stress-induced FADS1 expression in cancer cell growth, the 786-o and A498 cells were treated with different concentrations of an ER stress inducer Tunicamycin (TM; Cell Signaling Technology, Danvers, MA, USA) separately in two concentrations 1 ng/mL or 10 ng/mL, with DMSO as the vehicle control. The qPCR assay was used to evaluate the ER stress associated genes and FADS1 gene expression, which was shown in the “RNA extraction and RT-qPCR” section.

To investigate the function of FADS1 in ER stress-induced cancer cell apoptosis and reduced growth, 786-o and A498 cells were treated with the low-dose TM (1 ng/mL) separately for 18 and 24 hrs. In some experiments, 2000 nM D5D-IN-326 (DMSO as vehicle) was co-treated with the ER stress-induced cells for an additional 30 h (total 48 h). Cell apoptosis and proliferation were evaluated by the immunofluorescence assay, as described in the “Immunofluorescence staining” section.

### 2.6. Cell cycle assay

shRNA (sh-scramble or sh-FADS1) or D5D-IN-326 (DMSO as vehicle) treated 786-o and A498 cells were cultured in a 12-well plate for 96 h. The cells were washed in a cold PBS solution (Thermo Fisher Scientific, Waltham, MA, USA), followed by propidium iodide (PI, Abcam biotechnology company, Cambridge, UK) staining for 20 min at 37 °C. Flow cytometry (Microscopy, Imaging & Cytometry Resources core facility at Karmanos Cancer Institute, Wayne State University) was used to detect the PI staining intensity, which was used to evaluate the cell cycle distribution.

### 2.7. RNA extraction and RT-qPCR

The 786-o or A498 cells treated with shRNA (sh-scramble or sh-FADS1), or ER stress inducer were cultured in a 6-well plate for 48 h. RNA was extracted using the RNA mini kit (Qiagen, Hilden, Germany). cDNA was synthesized using the reverse transcription kit (Qiagen, Hilden, Germany) and quantitative RT-PCR was performed using gene-specific primers (see Supplemental Table 2 for details) and SYBR Green master mix (Thermo Fisher Scientific, Waltham, MA, USA). Negative delta cycle threshold (−Δ Ct) values were calculated by using the housekeeping gene (*PPIA*).

### 2.8. Immunofluorescence staining

shRNA (sh-scramble, sh-ATF3, or sh-FADS1), overexpression (OE-stuffer and OE-ATF3), D5D-IN-326 (DMSO as vehicle), PERK inhibitor & D5D-IN-326 (DMSO as vehicle), or ER stress inducer treated 786-o and A498 cells were cultured on glass coverslips for 96 h. Cells were fixed with 4 % paraformaldehyde (Fisher Scientific, Hampton, NH, USA) for 10 min at room temperature. Following PBS washing, fixed cells were incubated with PBS solution containing 5 % donkey serum (Jackson ImmunoResearch Labs, West Grove, PA, USA) and 0.2 % Triton X-100 (Thermo Fisher Scientific, Waltham, MA, USA) for 1 h at room temperature. Cells were then incubated with primary antibodies overnight at 4°C (the primary antibodies are listed in Supplemental Table 3). The following day, cells were incubated with secondary antibodies for 2 h at room temperature (the secondary antibodies are listed in the Supplemental Table 4). 4′,6-diamidino-2-phenylindole (DAPI; Thermo Fisher Scientific, Waltham, MA, USA) was used to label all nuclei. All samples were observed and imaged by Keyence microscope (Keyence, IL, USA). Low magnification images were used to check the total condition of the cells in each well, while high magnification images were used to assess protein expression.

### 2.9. Western blot

shRNA (sh-scramble or sh-FADS1), or D5D-IN-326 (DMSO as vehicle) treated 786-o and A498 cells were cultured in a 6-well plate for 0–48 h. Cells were lysed in RIPA buffer (Thermo Fisher Scientific, Waltham, MA, USA) which contained a complete protease inhibitor cocktail for 30 min at 4 °C. The resulting lysates were centrifuged at 12,000 g for 10 min and supernatants were collected. The protein concentration of each lysate was determined by the Pierce BCA protein assay kit (Thermo Fisher Scientific, Waltham, MA, USA). Cell lysates (20 μg) were analyzed via electrophoresis using 4 %−12 % sodium dodecyl sulphate-polyacrylamide gradient gels (Thermo Fisher Scientific, Waltham, MA, USA). Pre-stained protein ladders (Thermo Fisher Scientific, Waltham, MA, USA) were used to determine the protein size. Proteins were transferred to nitrocellulose membranes (Thermo Fisher Scientific, Waltham, MA, USA) and blocked with 5 % non-fat milk (Thermo Fisher Scientific, Waltham, MA, USA) in TBST (Thermo Fisher Scientific, Waltham, MA, USA) for 30 min at room temperature. Different primary antibodies were incubated with membranes over night at 4 °C (the primary antibodies are listed in the Supplemental Table 5). Following TBST washing, membranes were incubated with appropriate secondary antibodies for 1 hr at room temperature (the secondary antibodies are listed in Supplemental Table 6). The ChemiGlow West Chemiluminescence Substrate kit (Thermo Fisher Scientific, Waltham, MA, USA) was used to detect chemiluminescent signals using a ChemiDoc imaging system (Bio-Rad, CA, USA). ImageJ [24] (NIH, USA) was used to quantify protein bands on blot images. Housekeeping proteins (GAPDH or Vinculin) were used as an internal control to normalize the protein expression data.

### 2.10. Animal study

*In vivo* xenograft studies were performed on 7–8-weeks-old male and female SCID mice purchased from Charles River laboratories (Wilmington, MA, USA). All experiments involving mice were performed in accordance with the protocol approved by the Institutional Animal Investigational Committee of Wayne State University and NIH guidelines (IACUC-21–12–4269). 786-o cells were injected subcutaneously into both the right and left flanks of the mice, with the same mouse receiving both wild-type and sh-FADS1 cells in different flank to serve as an internal control. Cells in PBS were mixed 1:1 with Cultrex ^™^ (R&D systems, Minneapolis, MN, USA) at a final concentration of 1 × 10^6^ in 100 μl prior to injection. Control cells (N = 8) were injected in the right flank, sh-FADS1 treated cells (N = 8) were injected in the left flank. Two-weeks after injection, tumors were palpated and, if measurable, measured via caliper twice weekly. Mice were monitored for any signs of distress, including dehydration, weight loss or any loss in mobility. After 8 weeks (well before becoming overtly moribund), all mice were euthanized in accordance with AVMA guidelines. The tumors were resected and measured (weight and size), imaged, and either snap frozen in liquid nitrogen for RNA analysis, or fixed in Z-fix (Anatech, Battle Creek, MI, USA) for embedding and histology. All animal experiments complied with the ARRIVE guidelines and followed the National Research Council’s Guide for the Care of Use of Laboratory Animals.

### 2.11. Lipidomic analysis

786-o and A498 cells treated with sh-scramble or sh-FADS1 were further treated with 2 μM D5D-IN-326 (DMSO as vehicle) for 48 and 96 h. Subsequently, total phospholipids were separated on silica gel plates using 90: 8: 1: 0.8 (volume ratio) chloroform / methanol / acetic acid / water. The isolated total phospholipids were transesterified using 6 % methanolic hydrochloric acid over night at 80 °C. Gas chromatography-mass spectrometry was used to analyze the ratio of FADS1 substrate and product arachidonic acid/dihomo-γ-linolenic acid (AA/DGLA) according to previously established methods [25].

### 2.12. Metabolomic analysis

786-o sh-scramble and sh-FADS1 cells were seeded and cultured to 90 % confluency. The cells were rapidly (<15 s) rinsed twice with warm PBS to remove the culture medium, ensuring minimal leakage of intracellular metabolites. Subsequently, the entire cell culture dish was immersed in liquid nitrogen to quickly halt metabolic activity. Metabolites in frozen cells were extracted twice with 80 % methanol (pre-cooled at −80°C). Supernatant from two extractions were combined and dried in a CentriVap^®^ vacuum concentrator (Kansas City, MO) at 6°C. The dried cell extract was reconstituted and subject to LC-MS/MS based targeted metabolomic profiling of ~ 250 water soluble metabolites in the Pharmacology and Metabolomics Core at Karmanos Cancer Institute, as described previously [26]. Targeted metabolomics platform quantitate ~ 250 metabolites that are involved in the major human metabolic pathways, including central carbon metabolism (glycolysis, PPP and TCA), amino acid metabolism, nucleotide metabolism, etc. Central carbon metabolism is the main pathway for energy generation. Metabolite concentrations were normalized to protein concentration of each cell sample. Data analysis was carried out using the MetaboAnalyst software (https://www.metaboanalyst.ca/).

### 2.13. Quantification study and statistical analysis

All biological samples were collected from at least 3–6 independent experiments. The immunofluorescence intensity of nuclei in each well was analyzed by the ImageJ software (NIH, USA). DAPI, Ki-67, and cCASP3 positive cells were counted using the cell counter plugin of the ImageJ software. The percentage of positive cells were calculated as (number of the positive cells) *100 % / (number of the DAPI positive cells). For RT-qPCR, negative delta cycle threshold (-Δ Ct) values were calculated by using the housekeeping gene (*PPIA*). The 2^(- ΔΔ Ct) was used to determine the relative quantification of gene expression. For Western blots, ImageJ was used to quantify protein bands on each blot image. Housekeeping proteins (GAPDH or Vinculin) was used as an internal control to normalize the protein expression. For flow cytometry, flowJo software (BD, Franklin Lakes, NJ, USA) was used to quantify cell populations. The Dean-Jett-Fox algorithm [27,28] was used to model the cell cycle and calculate the proportion of each phase. For statistical analysis, data were analyzed using Graphpad Prism software (Boston, MA, USA), which included two-tailed, unpaired student’s t test or one way ANOVA test followed by Tukey comparison. P-values less than 0.05 were considered statistically significant. For comparisons, data were expressed as fold change relative to the control group. The figure’s diagram was generated using BioRender (Science Suite Inc, Toronto, ON, Canada).

## 3. Results

### 3.1. Inhibiting FADS1 activity suppresses renal cancer cell proliferation in vitro

**Fig. 1.**
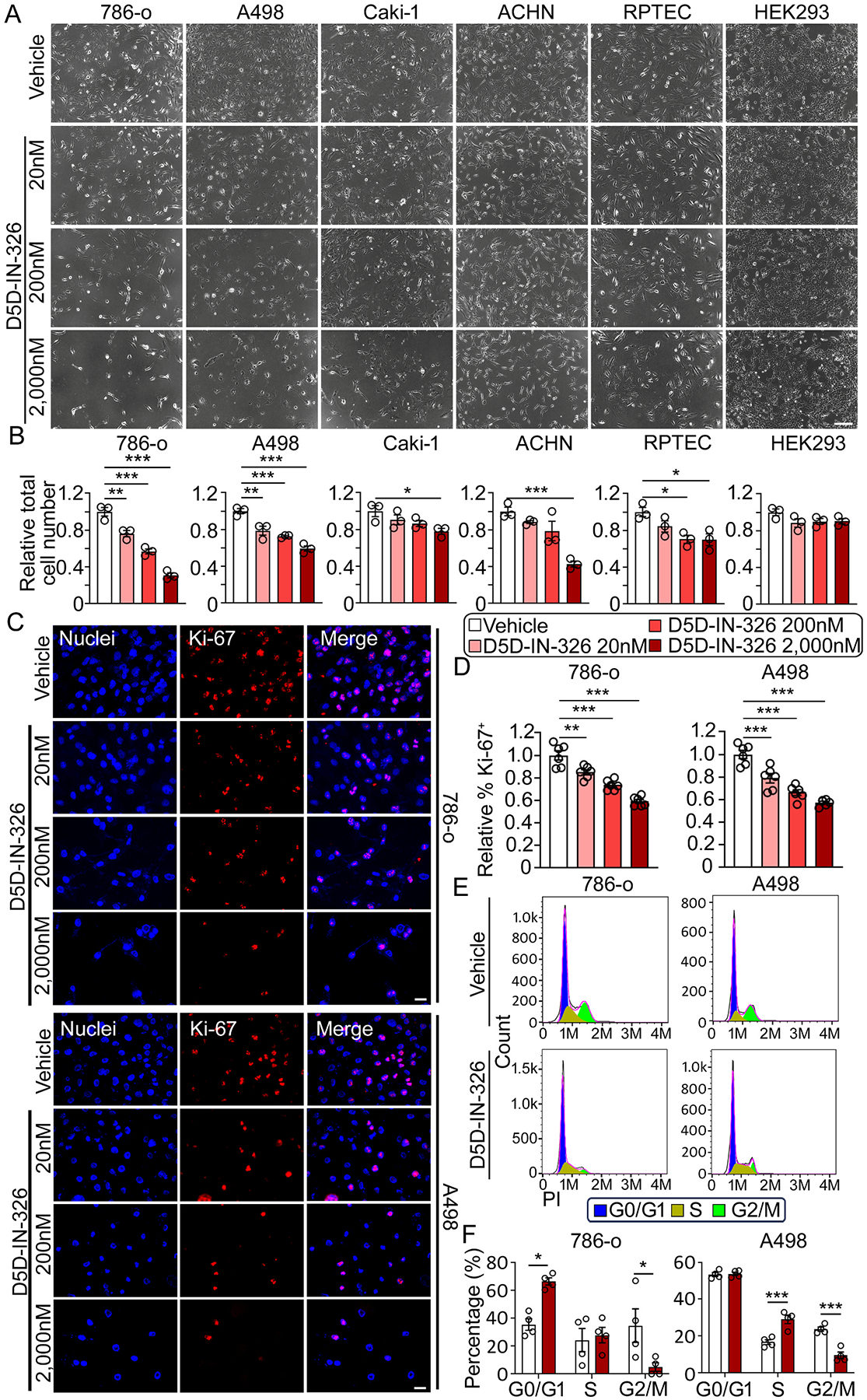
Inhibiting Fatty Acid Desaturase 1 (FADS1) suppressed human renal cancer cell growth *in vitro*. (A) Representative phase images of human renal cancer cells (786-o, A498, Caki-1, and ACHN) and human normal cells (RPTEC and HEK293) treated with increasing concentrations (vehicle, 20, 200, and 2000 nM) of D5D-IN-326 (FADS1 inhibitor) for 96 h. Scale bar: 200 μm. (B) The column bar graphs showing the relative quantification of total cell numbers following D5D-IN-326 (FADS1 inhibitor) treatment for 96 h (mean ± standard error, data were normalized to the vehicle group). Statistical analysis was conducted using Tukey’s multiple comparisons test. *P < 0.05; **P < 0.01; ***P < 0.001. (C) Representative immunofluorescence images illustrating Ki-67 expression in human renal cancer cells (786-o; top panels & A498; bottom panels) treated with increasing concentrations (vehicle, 20, 200, and 2000 nM) of D5D-IN-326 for 96 h. Scale bar: 10 μm. (D) The column bar graphs showing the relative quantification of the percentage of Ki-67 positive cells in human renal cancer cells (786-o; left & A498; right) subjected to gradient D5D-IN-326 treatment for 96 h (mean ± standard error). Data were shown as normalized ratios to the vehicle group. Statistical analysis conducted using Tukey’s multiple comparisons test. **P < 0.01; ***P < 0.001. (E) Representative histogram showing the expression of the propidium iodide (PI) in human renal cancer cells (786-o; left & A498; right) subjected to gradient D5D-IN-326 treatment for 96 h. The different stages of cell cycle (G0/G1, S, and G2/M stage) were determined by the expression of PI, as shown in the plot. (F) The column bar graphs showing the percentage of cells (786-o; left & A498; right) distributed in each cell cycle stage (G0/G1, S, and G2/M) after the D5D-IN-326 treatment for 96 h (mean ± standard error). A gradient increase in the shape of the red bars was used to represent the increasing concentration of D5D-IN-326 treatment in panels B, D, and F, ensuring uniform representation across the entire figure. Statistical analysis conducted using two-tailed unpaired Student’s t test. *P < 0.05; ***P < 0.001.

In this study, we utilized a recently discovered small molecule FADS1 inhibitor D5D-IN-326 to inhibit FADS1 activity. This inhibitor has demonstrated selectivity for FADS1 in previous studies [7]. Treatment with D5D-IN-326 significantly inhibits the conversion of dihomo-γ-linolenic acid (DGLA), the direct substrate of FADS1, into arachidonic acid (AA), as indicated by the reduced AA/DLGA ratio in the renal cancer cell line 786-o and A498 at 48 h and 96 h (Supplementary Figure 1). To investigate the impact of FADS1 inhibition on renal cancer cell growth, we treated human embryonic kidney cell line (HEK293), human renal proximal tubule epithelial cells (RPTEC), and various renal cancer cell lines (786-o, A498, ACHN, and Caki-1) with increasing concentrations of D5D-IN-326 for 96 h (Fig. 1A). The results show that D5D-IN-326 treatment significantly reduced the number of renal cancer cells, while it has minimal effect on normal RPTEC renal cells or HEK293 cells (Fig. 1B). We chose 786-o and A498 cells for further evaluation, since both are classified as typical primary ccRCC cells and widely accepted as models of ccRCC, while ACHN and Caki-1 are derived from metastatic tumors [29].

To investigate whether FADS1 inhibition affects cell proliferation, we performed immunofluorescence staining of proliferation marker Ki-67 in 786-o and A498 cells treated with increasing concentrations of the FADS1 inhibitor for 96 hrs. The data revealed a significant, dose-dependent decrease in the percentage of Ki-67 positive (Ki-67^+^) cells upon FADS1 inhibitor treatment compared to vehicle control (Fig. 1C & 1D). Additionally, cell cycle analysis showed that a significant fraction of 786-o cells treated with the FADS1 inhibitor (2000 nM) was arrested in the G0/G1 stage, while fewer cells were arrested in the G2/M stage. In addition, A498 cells treated with the FADS1 inhibitor (2000 nM) were arrested in the S stage, with fewer cells arrested in the G2/M stage (Fig. 1E & 1F). Collectively, these results indicate that FADS1 inhibition reduces renal cancer cell proliferation by inducing cell cycle arrest at the interphase stages preparing cell growth and DNA synthesis prior to cell mitosis.

### 3.2. Renal cancer cell proliferation is inhibited by FADS1 knockdown

**Fig. 2.**
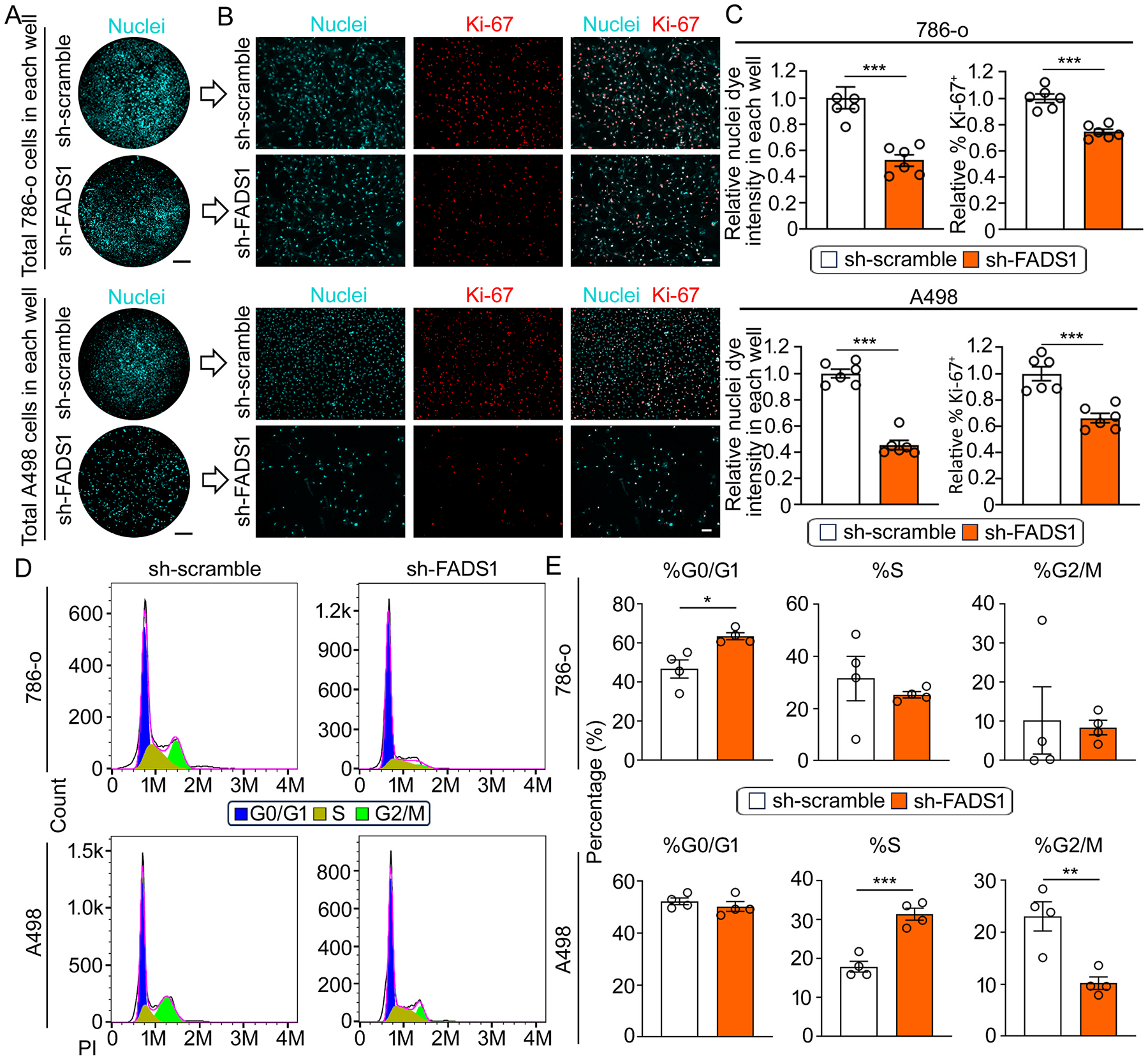
Suppression of *FADS1* gene inhibited human renal cancer cell growth. (A) Representative overview images of nuclei staining depict *FADS1* knockdown (sh-FADS1; bottom) and control (sh-scramble; top) 786-o (top) and A498 (bottom) cells in each well. Scale bar: 10 mm. (B) Representative immunofluorescence images showing expression of Ki-67 in sh-scramble and sh-FADS1 786-o (top) or A498 (bottom) cells. Scale bar: 50 μm. (C) The column bar graphs showing the relative quantification of whole nuclei staining intensity and the percentage of Ki-67 positive cells in sh-scramble and sh-FADS1 786-o or A498 cells (mean ± standard error). Data were normalized to the sh-scramble group. Statistical analysis performed using two-tailed unpaired Student’s t test. ***P < 0.001. (D) Representative histogram showing the expression of the propidium iodide (PI) in sh-scramble and sh-FADS1 786-o (top) or A498 (bottom) cells. The different stages of cell cycle (G0/G1, S, G2/M stage) were determined by the expression of PI, as shown in the plot. (E) The column bar graphs showing the relative quantification of the percentage of each cell cycle stage (G0/G1, S, and G2/M) in sh-scramble and sh-FADS1 786-o (top) or A498 (bottom) cells (mean ± standard error). Data were normalized to the sh-scramble group. Statistical analysis conducted using two-tailed unpaired Student’s t test. *P < 0.05; **P < 0.01; ***P < 0.001.

To further verify the impact of FADS1 inhibition on RCC cell proliferation and to elucidate the role of FADS1 in renal cancer cell growth, we employed FADS1-specific shRNA to knock down FADS1 expression in 786-o and A498 cells. FADS1 mRNA and protein expression in sh-FADS1 786-o and A498 cells were significantly reduced compared to the cells treated with scrambled shRNAs (Supplementary Figure 2 & 3). As expected, FADS1-knockdown (FADS1-KD or sh-FADS1) significantly reduced the ratio of AA/DGLA in the renal cancer cell line 786-o (Supplementary Figure 4). Consequently, the total number of FADS1-KD 786-o and A498 cells was significantly lower than the scramble shRNA-treated cells, and the percentage of Ki-67^+^ cells was also significantly reduced (Fig. 2A, 2B, & 2C). Moreover, cell cycle analysis revealed that FADS1 knockdown led to cell cycle arrest at the G0/G1 stage in 786-o cells and at the S stage in A498 cells (Fig. 2D & 2E), mirroring the effects observed with FADS1 inhibitor treatment. These findings confirm the growth suppressing effects of pharmacological FADS1 inhibition and underscore the crucial role of FADS1 gene expression in renal cancer cell growth.

### 3.3. Inhibition of FADS1 induces ER stress in renal cancer cells

**Fig. 3.**
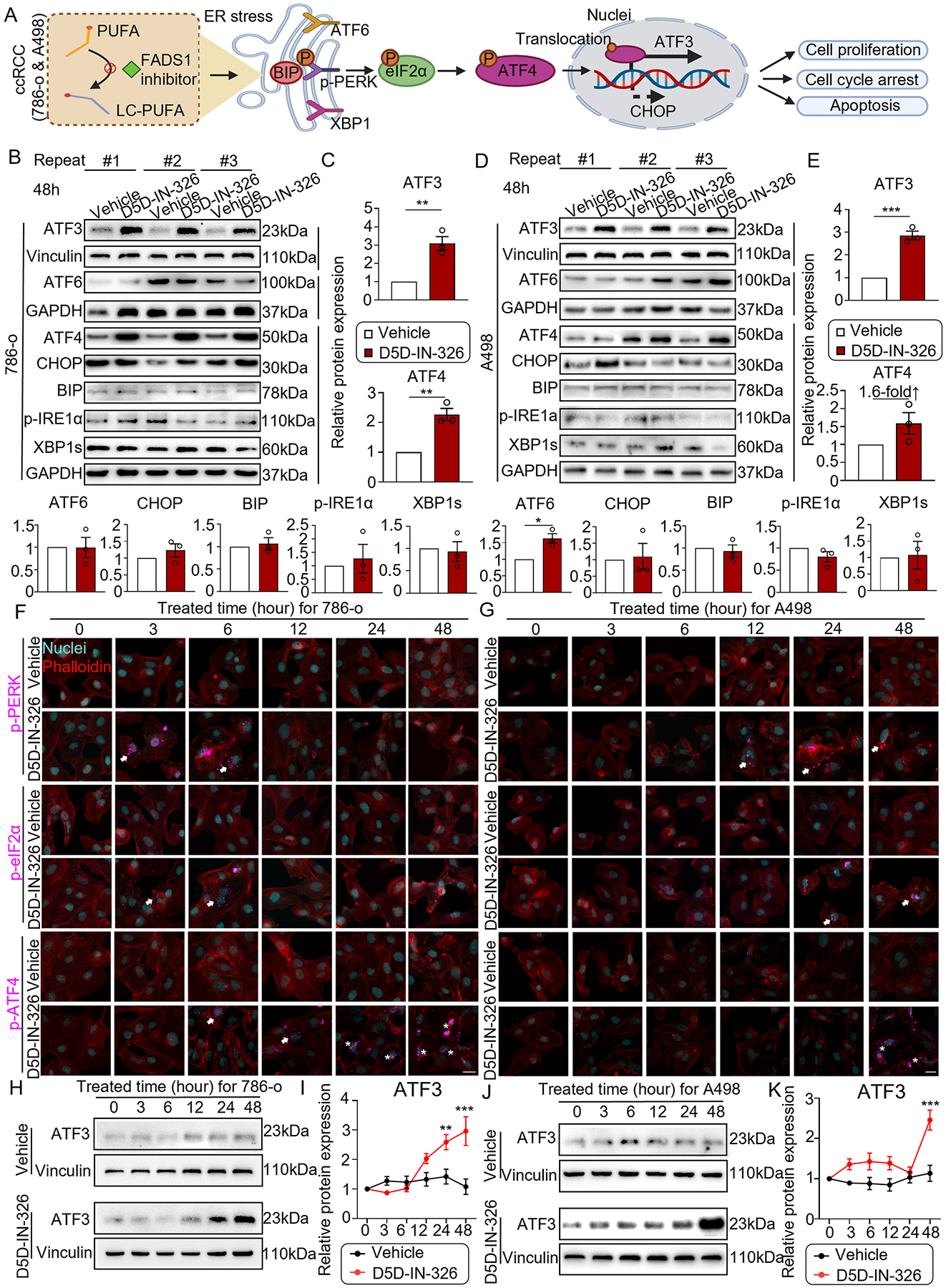
FADS1 and ER stress in human renal cancer cells. (A) Diagram representing the associated ER stress pathway in the FADS1 inhibitor triggered the activation in the human renal cancer cells (786-o and A498). (B-E) Western blot images illustrating the expression of ER stress-associated proteins (ATF3, ATF6, ATF4, CHOP, BIP, pIRE1α, and XBP1s; Vinculin or GAPDH as housekeeping protein) in 786-o and A498 cells treated with 2000 nM D5D-IN-326 (vehicle as control) for 48 h. Showing here are results of assays independently repeated for three times. The column bar graphs showing the relative quantification of ER stress-associated proteins in 786-o (C) and A498 (E) cells treated with 2000 nM D5D-IN-326 (vehicle as control) for 48 h (mean ± standard error). Each ATF3 and FADS1 protein level was normalized to its corresponding sh-scramble group in each experimental replicate. Consequently, all sh-scramble values were set to ‘1,’ with no variability. Statistical analysis performed using two-tailed unpaired Student’s t test. *P < 0.05; **P < 0.01. (F) The representative fluorescence images showing the expression of the p-PERK, p-eIF2α, and p-ATF4 staining in the 786-o and A498 (G) cells treated with 2000 nM D5D-IN-326 (vehicle as control) for 0, 3, 6, 12, 24, and 48 h (cells were labelled with DAPI and phalloidin; arrows showed the positive signal and stars showed the specific protein translocated into the cell nuclei). Scar bar: 10 μm. (H) Represented Western blot images illustrating the expression of ATF3 (Vinculin as housekeeping protein) in 786-o and A498 (J) cells treated with 2000 nM D5D-IN-326 (vehicle as control) for 0, 3, 6, 12, 24, 48 h. (I) The column bar graphs show the relative quantification of ATF3 in 786-o and A498 (K) cells treated with 2000 nM D5D-IN-326 (vehicle as control) for 0–48 h (mean ± standard error). Data were normalized to the vehicle group. Statistical analysis performed using two-way ANOVA test followed with multiply comparison. *P < 0.05; **P < 0.01.

PUFAs are typically integrated into phospholipids in cells and play an important role in various functions of the cell membrane system. Previous studies have demonstrated that lipid desaturation is crucial for maintaining ER homeostasis. Inhibiting the desaturation process in the production of MUFAs leads to ER stress [30–33]. Thus, we hypothesized that inhibition of PUFA desaturation by targeting FADS1 would also induce ER stress and subsequently hinder the cancer cell growth. There are three main pathways that can trigger ER stress, including the PERK, ATF6, and XBP1 pathways ([34], Fig. 3A). To evaluate which specific pathway is associated with the effect of FADS1 inhibition, the expression of all ER stress-associated proteins was screened in FADS1 inhibitor-treated groups in 786-o and A498 cells. Our data showed that assessing the expression of ER stress-related proteins in 786-o and A498 cells with or without FADS1 inhibitor treatment revealed a significant increase in the protein expression of both ATF3 and ATF4 in FADS1 inhibitor-treated 786-o and A498 cells (Fig. 3B–E). A498 also showed a moderate activation of ATF6. Other ER stress markers did not show significant difference. This suggests that the inhibition of FADS1 may primarily activate the ATF4-ATF3 pathway to exert associated functions.

To further validate this notion, we evaluated the activation of ATF4 and its upstream proteins, including p-PERK and p-eIF2α. Our data indicated a time-dependent activation of this signaling with the expression of both p-PERK and p-eIF2α shown to be induced after 3–6 h of FADS1 inhibitor treatment in 786-o cells. Activation of ATF4 requires protein phosphorylation and translocation of the phosphorylated ATF4 (p-ATF4) into the cell nuclei [35–37]. We found that p-ATF4 expression and translocation were detected starting from 6 h and persisted up to 48 h (Fig. 3F). In A498 cells, both p-PERK and p-eIF2α were expressed after 24 h of FADS1 inhibitor treatment, while p-ATF4 expression and nuclear translocation occurred at 48 h of FADS1 inhibitor treatment (Fig. 3G). Additional assays demonstrated that p-PERK, p- eIF2α, and p-ATF4 remained expressed in A498 cells after 72 h of D5D-IN-326 treatment (Supplementary Figure 5). The expression of ATF3 significantly increased after 24 and 48 h of FADS1 inhibitor treatment in 786-o and A498 cells, respectively (Fig. 3H–K). Taken together, the inhibition of FADS1 in renal cancer cells promoted ER stress via activating the PERK/eIF2α/ATF4 pathway, with ATF3 likely playing a key role in mediating this effect.

### 3.4. PERK inhibition rescued the FADS1 inhibitor-induced suppression for cell proliferation

**Fig. 4.**
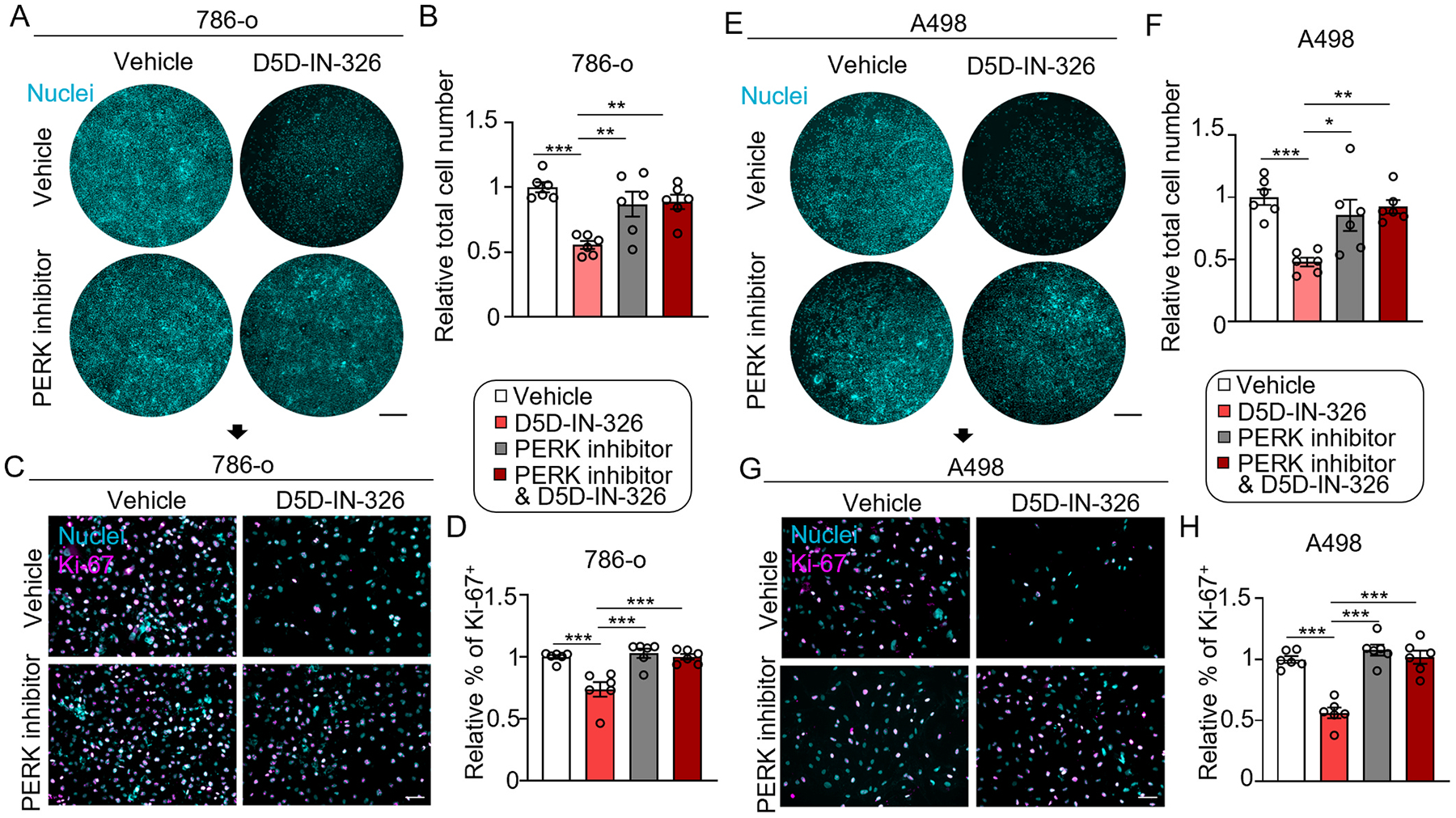
PERK inhibition rescued FADS1 inhibitor-induced cell proliferation. (A) Representative images of nuclei staining showing 786-o cells treated with 2000 nM D5D-IN-326 or 50 nM PERK inhibitor in each well (A498 cells for E). Scale bar: 10 mm. (B) Relative quantification of whole nuclei staining intensity in the 786-o (A498 cells for F; mean ± standard error). Data were normalized to the vehicle group. Statistical analysis conducted using Tukey’s multiple comparisons test. **P < 0.01; ***P < 0.001. (C) Representative immunofluorescence images illustrating expression of Ki-67 in cells treated with 2000 nM D5D-IN-326 or 50 nM PERK inhibitor in 786-o (A498 cells for G). Scale bar: 50 μm. (D) Relative quantification of percentage of Ki-67 positive cells in 786-o (A498 cells for H; mean ± standard error). Data were normalized to the vehicle group. Statistical analysis conducted using Tukey’s multiple comparisons test. ***P < 0.001.

To verify whether the cell proliferation suppression induced by FADS1 inhibition is causally mediated by the PERK-driven ER stress, we examined the impact of FADS1 inhibitor on cell proliferation with and without co-treatment with a PERK inhibitor in both 786-o and A498 cells. As demonstrated in Fig. 4A–H, while FADS1 inhibitor treatment significantly reduced cell number and Ki-67^+^ cells, PERK inhibitor co-treatment rescued such an impact in both cell lines. Together with the data presented in the section above, this observation suggests that the FADS1 inhibition-induced cell proliferation is mediated by the PERK-driven ER stress signaling.

### 3.5. FADS1 plays critical role in protecting renal cancer cells from persistent ER stress

**Fig. 5.**
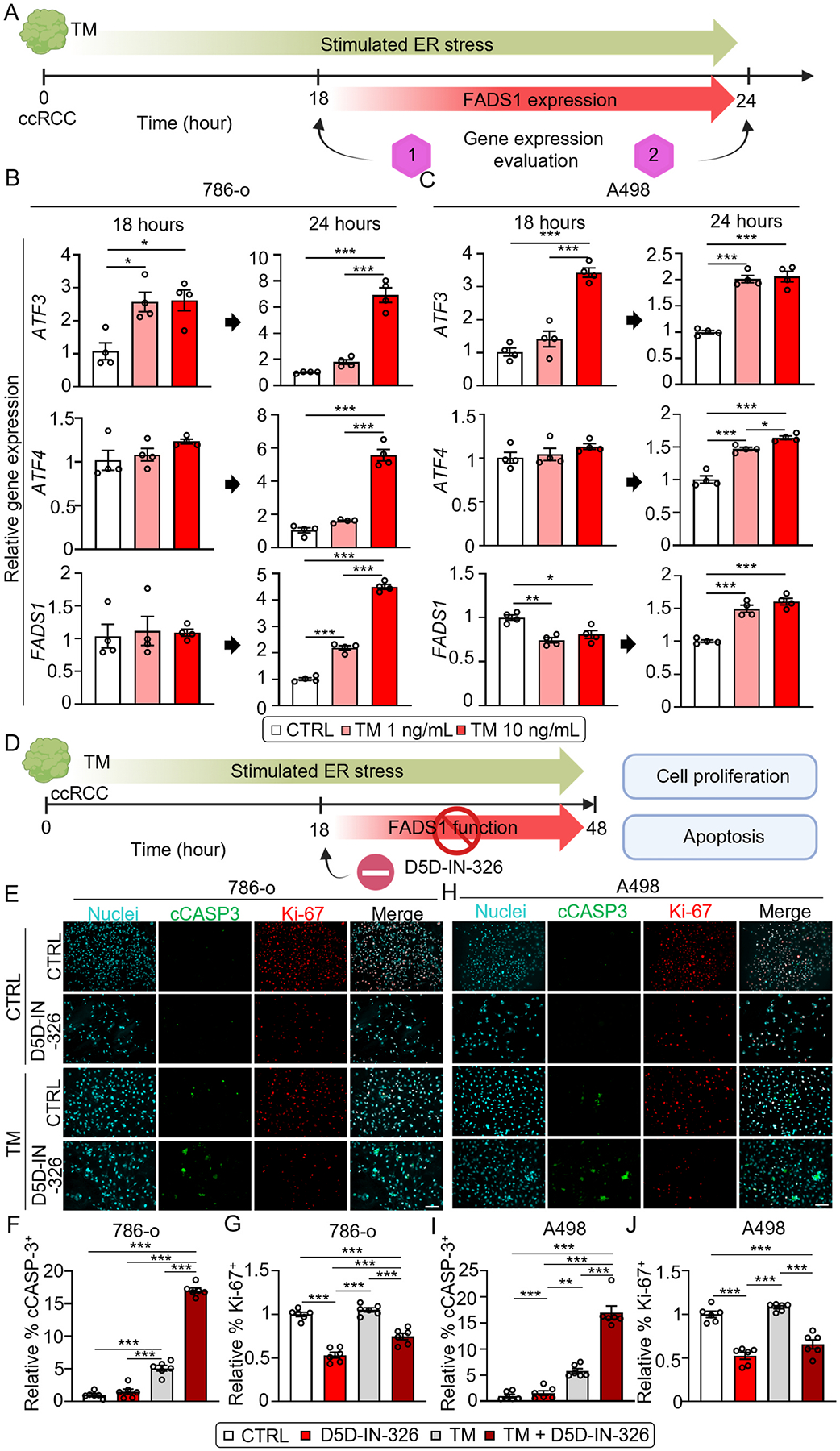
FADS1 is necessary to rescue cells from persistent ER stress. (A) Diagram showing the tunicamycin (TM) treated 786-o and A498 for 24 h. The mRNA levels of *ATF3, ATF4* and *FADS1* gene were evaluated at 18 and 24 h post treated. (B) The column bar graphs illustrating the relative gene expression levels of ER stress-associated genes (*ATF3* and *ATF4*) and *FADS1* in 786-o and A498 (C) cells treated with gradient concentrations of TM (vehicle, 1 ng/mL, and 10 ng/mL) for 18 h and 24 h (mean ± standard error). Data were normalized to the vehicle group. Statistical analysis was conducted using Tukey’s multiple comparisons test. *P < 0.05; **P < 0.01; ***P < 0.001. (D) Diagram showing the TM treatment design for total 48 h to examine the role of FADS1 in persistent ER stress. Cells were continuously treated with TM (1 ng/mL) for 48 hrs. At 18 h, cells were further treated with 2000 nM FADS1 inhibitor (D5D-IN-326) or vehicle for additional 30 hrs (total 48 hrs). The cell proliferation and apoptosis were evaluated at the 48 h. (E) Representative immunofluorescence images displaying expression of cCASP3 and Ki-67 in 786-o and A498 (H) cells treated with 2000 nM D5D-IN-326 (vehicle as control) for 30 h, with or without 18-h pre-treatment with 1 ng/mL TM. Scale bar: 100 μm. Bar graphs showing the relative quantification of the percentage of cCASP3-positive cells (F for 786-o & I for A498) and Ki-67 positive cells (G for 786-o & J for A498) human renal cancer cells (786-o & A498) with or without TM pre-treatment subjected to D5D-IN-326 treatment (vehicle as control) for 48 h (mean ± standard error). Data were normalized to the vehicle group. Statistical analysis conducted using Tukey’s multiple comparisons test. **P < 0.01; ***P < 0.001.

In order to further explore the relationship between FADS1 and ER stress, we treated cells with an ER stress inducer, tunicamycin (TM), to trigger ER stress response in wild type 786-o and A498 cells. After 18 hrs of treatment with TM, our data showed that the transcription of the aforementioned ER stress markers (*ATF3* and *ATF4*) started to be activated, though the pattern of such an induction in the two cell lines is slightly different. However, FADS1 expression remains unchanged. After 24 h of treatment with TM, transcription of *ATF3, ATF4* and *FADS1* were all significantly increased in both cells (Fig. 5A–C). This data suggests that while *FADS1* transcription may not be responsive to acute ER stress, it is activated by extended ER stress in renal cancer cells.

Cancer cells often experience various extrinsic and intrinsic perturbations which lead to ER stress [38,39]. Kidney cancers are known to be sensitive to hypoxia which induces ER stress [40,41]. Mild ER stress typically results in activation of signaling pathways that rescue cancer cells from stressful conditions. However, extreme or persistent ER stress may be unresolvable, which can lead to cell cycle arrest and apoptosis [42–44]. To determine if FADS1 is critical for protection of cancer cells from extreme ER stress and the maintenance of cancer cell survival and growth, we pretreated 786-o and A498 cells with TM (1 ng/mL) for 18 hrs. Subsequently, cells were co-treated with both TM and the FADS1 inhibitor (2000 nM) for additional 30 hrs (48 hrs for total). The co-treatment resulted in a significant increase in cell apoptosis, characterized by the significant elevation of cCASP3-positive cells in TM-treated 786-o and A498 cells compared to cells with TM or D5D-IN-326 treatment alone (Fig. 5E–J). Furthermore, FADS1 inhibition led to decreased number of Ki-67^+^ cells, though co-treatment with TM did not show a significant synergistic effect, possibly due to that TM alone at this concentration does not exhibit impact on cell proliferation during the timeframe or the limitation of using Ki-67 to reflect the complete profile of cell proliferation. These findings suggest that FADS1 function is critical for maintaining ER homeostasis following the induction of ER stress, and inhibiting FADS1 sensitizes cellular response to ER stress, inducing cell apoptosis.

### 3.6. FADS1 regulation of renal cancer cell proliferation depends on ATF3

**Fig. 6.**
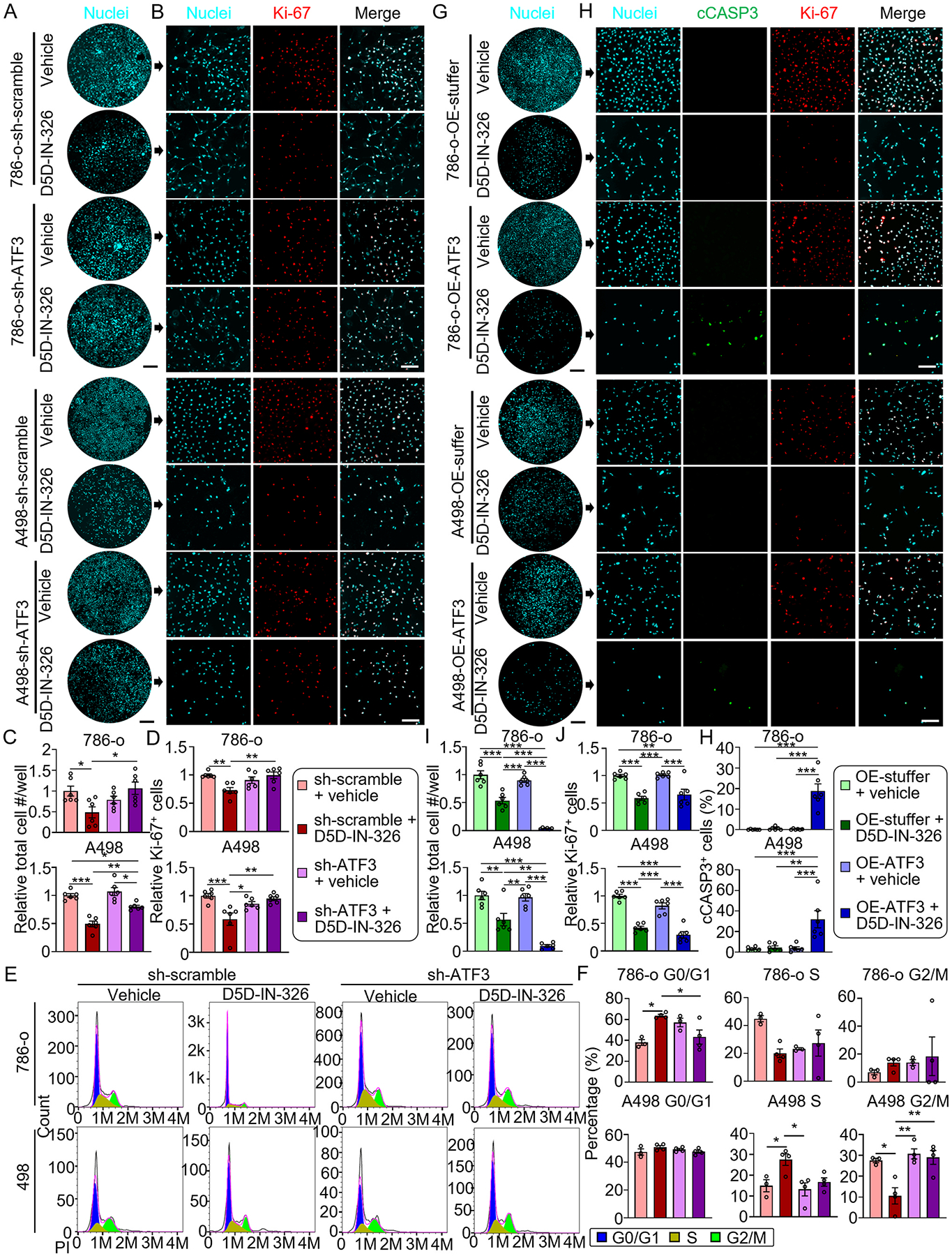
ATF3 mediates the impact of FADS1 inhibition on cell proliferation suppression and cell cycle arrest. (A) Representative images of nuclei staining showing *ATF3* knockdown (sh-ATF3) in 786-o (top) or A498 (bottom) cells treated with D5D-IN-326 (sh-scramble or sh-ATF3 with vehicle or D5D-IN-326) in each well. Scale bar: 10 mm. (B) Representative immunofluorescence images illustrating expression of Ki-67 in cells treated with sh-scramble with vehicle, sh-scramble with D5D-IN-326, sh-ATF3 with vehicle, and sh-ATF3 with D5D-IN-326 in 786-o (top) or A498 (bottom) cells. Scale bar: 100 μm. (C) Relative quantification of whole nuclei staining intensity and (D) the percentage of Ki-67 positive 786-o (top) and A498 (bottom) (mean ± standard error). Data were normalized to the sh-scramble+vehicle group. Statistical analysis conducted using Tukey’s multiple comparisons test. *P < 0.05; **P < 0.01; ***P < 0.001. (E) Representative histogram showing the intensity of the propidium iodide (PI) staining in sh-scramble and sh-FADS1 treated 786-o (top graphs) or A498 (bottom graphs) cells with or without D5D-IN-326 treatment. The different stages of cell cycle (G0/G1, S, G2/M stage) were determined by the intensity of PI, as shown in the plot. (F) The column bar graphs showing the quantification of the percentage of each cell cycle stages (G0/G1, S, and G2/M) in cells treated with sh-scramble with vehicle, sh-scramble with D5D-IN-326, sh-ATF3 with vehicle, and sh-ATF3 with D5D-IN-326 of 786-o (top graphs) or A498 (bottom graphs) cells (mean ± standard error). Statistical analysis conducted using one-way ANOVA followed by Tukey’s multiple comparisons test. *P < 0.05; **P < 0.01. (G) Representative images of nuclei staining showing *ATF3* overexpression (OE-ATF3) in 786-o (top) or A498 (bottom) cells treated with D5D-IN-326 (OE-stuffer or OE-ATF3 with vehicle or D5D-IN-326) in each well. Scale bar: 10 mm. (I) Representative immunofluorescence images illustrating whole nuclei staining intensity treated with OE-stuffer with vehicle, OE-stuffer with D5D-IN-326, OE-ATF3 with vehicle, and OE-ATF3 with D5D-IN-326 in 786-o (top) or A498 (bottom) cells. Scale bar: 100 μm. (J) Relative quantification of intensity expression of Ki-67 and (H) the percentage of cCASP3 positive 786-o (top) or A498 (bottom) cells (mean ± standard error). Data were normalized to the OE-stuffer + vehicle group. Statistical analysis conducted using Tukey’s multiple comparisons test. *P < 0.05; **P < 0.01; ***P < 0.001.

Given the significant increase in ATF3 expression in response to FADS1 inhibition, we set out to further investigate the potential role of ATF3 in mediating the impact of FADS1 inhibition on cancer cell growth. For this purpose, ATF3-specific shRNA to knock down ATF3 in 786-o and A498 cells were used. After optimizing ATF3 knockdown in these models (Supplementary Figure 2 & 3), cell growth and proliferation following FADS1 inhibitor treatment was examined. The total cell number and the percentage of Ki-67^+^ cells were significantly lower in FADS1 inhibitor-treated 786-o and A498 cells compared to untreated control. However, upon ATF3 knockdown (ATF3-KD or sh-ATF3), proliferation was restored with the treatment of FADS1 inhibitor (Fig. 6A–D). We further conducted cell cycle arrest assays to determine if ATF3 played a role in FADS1 inhibition-induced cell cycle arrest. Flow cytometry analysis indicated that FADS1 inhibition-induced cell cycle arrest was rescued, at least in part, by ATF3 gene knockdown (Fig. 6E & 6F). Using shRNA to knockdown both ATF3 and FADS1 instead of FADS1 inhibitor demonstrated a similar effect in both cells (Supplementary Figure 6).

Besides, we also overexpressed (OE) ATF3 in 786-o and A498 cells. We found that substantial ATF3 OE led to rapid cell death (Supplementary Figure 7). We then optimized the model by assuring a moderate ATF3 OE in the cells (Supplementary Figure 8). Our data showed that while the moderate ATF3 OE alone did not result in significant alterations in cell proliferation or cell death in both cells, total cell number was dramatically reduced and the percentage of cCAPS3^+^ cells were significantly increased when OE-ATF3 786-o or A498 cells were treated with FADS1 inhibitor (Fig. 6G–J). Taken together, our findings suggest that ATF3 plays a critical role in mediating the effect of FADS1 inhibition on ER stress, cell cycle, proliferation, and cell death.

### 3.7. FADS1 inhibition decreases biosynthesis of nucleotides and UDP-N-Acetylglucosamine

To further understand the potential metabolic remodeling that FADS1 inhibition may cause, we conducted metabolomic analyses using 786-o cells treated with sh-FADS1 or the scramble shRNA control. As a proof-of-concept for establishing the role of FADS1 in lipid metabolism, we noted that FADS1 knockdown significantly impacted pathways involving fatty acid metabolism, including biosynthesis, elongation, and degradation, which were characterized by significantly reduced levels of acetyl-CoA and CoA. Interestingly, pathways regulating amino sugar and nucleotide sugar metabolism were also enriched (Supplementary Figure 9A). Notably, FADS1-knockdown significantly reduced the level of nucleotides and their precursors, e.g., dATP, dGTP, dTDP, AICAR (5-aminoimidazole-4-carboxamide-1-β-D-ribofuranoside, a key metabolite involved in purine de novo biosynthesis), etc. More importantly, FADS1-knockdown significantly downregulated the level of UPD-N-Acetylglucosamine (UDP-GlcNAc), a critical substrate of protein glycosylation in the ER (Supplementary Figure 9B). Reduced UDP-GlcNAc has been shown to induce protein unfolding or misfolding, activating the unfolded protein response, a key step in ER stress homeostasis [45]. These findings suggest that FADS1 inhibition exerts a pronounced impact on nucleotides biosynthesis and protein folding, which may account for the cell cycle arrest and ER stress observed in our experiments.

### 3.8. FADS1 knockdown inhibits tumor growth in vivo

**Fig. 7.**
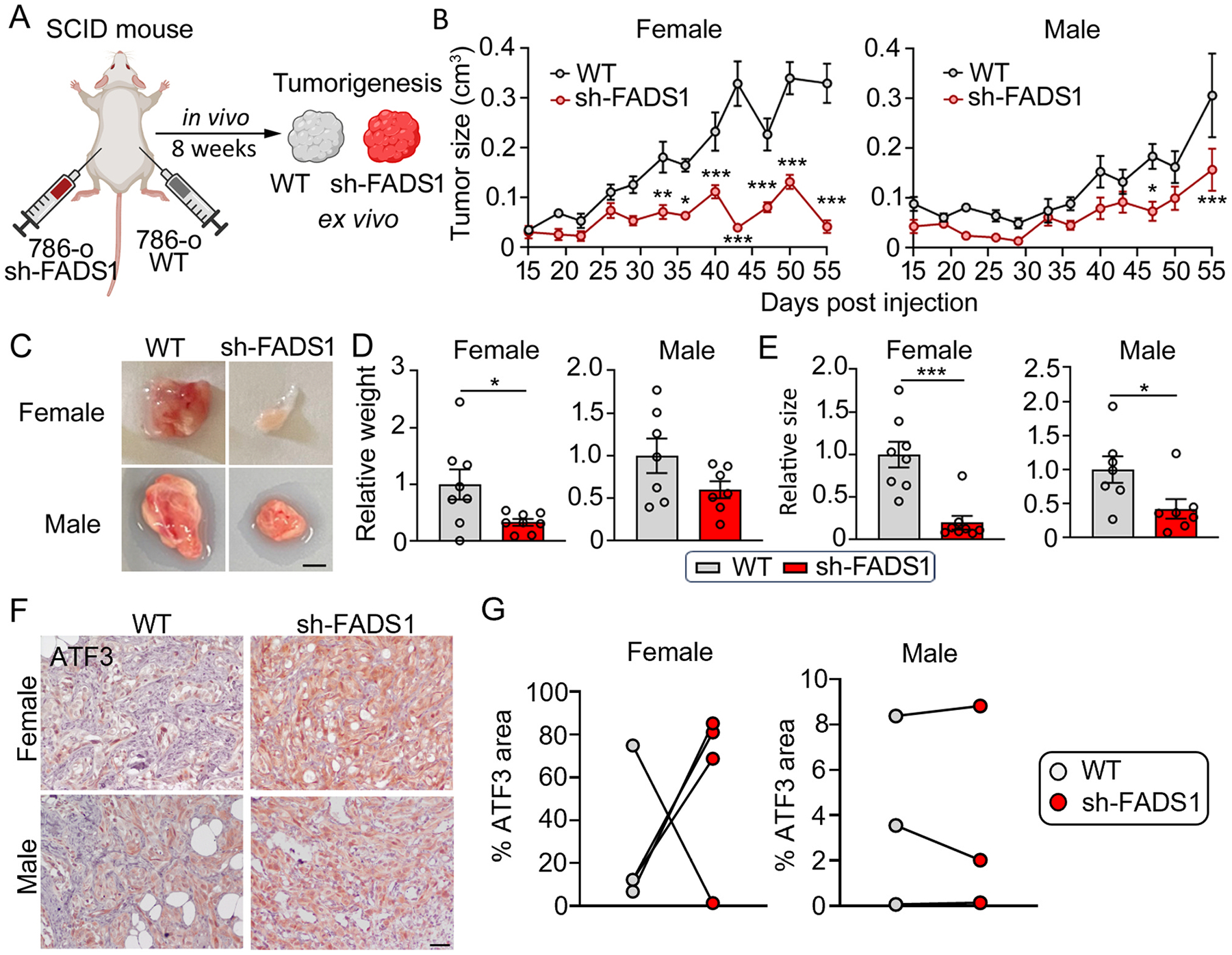
FADS1 knockdown reduces renal tumor growth *in vivo*. (A)Experimental diagram illustrating the human renal cancer cell xenograft study. FADS1 knockdown (sh-FADS1) 786-o cells were injected into the left flank of SCID mice, while wild type (WT) 786-o cells were injected into the right flank. Tumor growth was monitored for 8 weeks. (B) The graphs depicting average tumor size (cm^3^) of sh-FADS1 786-o cells and WT 786-o cells in female and male severe combined immunodeficiency disease (SCID) mice up to 55 days post injection (mean ± standard error). Statistical analysis performed using two-tailed unpaired Student’s t test. *P < 0.05; **P < 0.01; ***P < 0.001. (C) Representative images of extracted subcutaneous tumors from male and female mice. Scale bar: 0.25 cm. (D) Relative tumor weight (grams) and (e) size (cm^3^) in WT and KD subcutaneous tumors post extraction (mean ± standard error). Data were normalized to the WT group. Statistical analysis performed using two-tailed unpaired Student’s t test. *P < 0.05; ***P < 0.001. (F) Representative immunohistochemistry images showing the expression of ATF3 in the female and male tumors section. Scale bar: 50 μm. (G) The paired graphs depicting the percentage of ATF in each section. Statistical analysis performed using two-tailed paired Student’s t test.

To assess the impact of FADS1 on tumor development *in vivo*, we injected wild-type (WT) and FADS1-KD 786-o cells (Supplementary Figure 10A) into the flank of male and female severe combined immunodeficient (SCID) mice to generate tumors (Fig. 7A). We monitored tumorigenesis over a course of 55 days. The size of FADS1-KD tumors was significantly smaller than those formed by wild-type cells, both in female and male mice, with more significant difference in female mice (Fig. 7B). After 55 days, tumors were collected for further analyses (Fig. 7C; Supplementary Figure 10B). The weight of tumor xenografts derived from the FADS1-KD or control cells demonstrated a similar trend as the tumor size (Fig. 7D & 7E). To evaluate whether ATF3 is also a key factor in FADS1 inhibition-induced tumor growth restriction *in vivo*, immunochemistry staining was performed on tumor sections. Our data showed a trend of increased ATF3 expression in the tumors derived from sh-FADS1 cells (3 out of samples in females and 2 out 3 samples in males), though there was no statistical significance, possibly due to the small sample size (Fig. 7F–G). Taken together, these results demonstrated that reduced FADS1 function suppressed tumor formation *in vivo*, suggesting that FADS1 inhibiting might have a therapeutic potential for RCC.

## 4. Discussion

Increasing evidence suggests that FADS1 activity and PUFA metabolism are closely linked to cancer initiation, progression and metastasis [17]. Our previous research has demonstrated that increased FADS1 gene expression is significantly correlated with reduced cancer patient survival among multiple types of cancers, and especially in kidney cancer patients [7]. We also found that in kidney cancers, FADS1 mRNA levels demonstrated a significantly gradient elevation from adjacent normal tissue, primary RCC tumors, metastatic tumors to recurrent tumors [7], highlighting its potential role in cancer biology and disease progression. These early observations prompted us to further explore the causal role of FADS1 in RCC biology as well as the potential underlying mechanism. In this study, we demonstrate that FADS1 knockdown or pharmacological inhibition suppresses cell proliferation, alters cell cycle in RCC cells, and reduces tumor formation *in vivo*. We further provide evidence that FADS1 is essential for supporting cancer cell growth by protecting cancer cells from persistent ER stress, which is mediated by the PERK-ATF4 pathway with the involvement of a key transcription factor ATF3. Our data provide new knowledge regarding the essential role of FADS1 in RCC, supporting FADS1 as new target for renal cancer.

We found that FADS1 inhibition-induced suppression of cell proliferation is mediated by ER stress. The ER plays a pivotal role as a central organelle responsible for protein synthesis, folding, and maturation [46]. When cells are under stress, misfolded or unfolded proteins accumulate, which triggers the unfolded protein response (UPR) and initiates ER stress [46]. Cancer cells are often exposed to stressful microenvironmental conditions e.g. hypoxia and nutrient deprivation, as well as intrinsic conditions such as rapid metabolism and overproduction of reactive oxygen species (ROS), which typically lead to the activation of ER stress [47–49]. ER stress responsive signaling may activate downstream pathways to support tumor growth, metastasis, and adaptation to anti-cancer treatments [38,39,50]. However, extreme or persistent conditions may induce unresolvable ER stress, leading to cell death. There is a reciprocal relationship between lipids metabolism and ER homeostasis. While ER stress can disturb lipid synthesis, metabolism and intracellular trafficking, lipid supplementation and intracellular metabolism can also alter ER function and related signaling [51–53]. Previous studies have demonstrated the important role of fatty acid desaturation in maintaining ER homeostasis and function. Increased lipids saturation, augmenting levels of saturated fatty acids, induces ER stress [54–58], which has been attributed to a reduction in ER membrane fluidity [59], ER Ca(2^+^) depletion [60,61], overload of misfolded proteins [55], and direct interaction between lipids and ER signal proteins [57]. Notably, limiting MUFA biosynthesis by blocking SCD1 has also been shown to activate ER stress and cell death in ccRCC and other cancers [62–65]. FADS1 is a rate-limiting enzyme responsible for delta-5 desaturation of both omega-3 and omega-6 LC-PUFAs, with its immediate metabolic products being AA and EPA. Our study demonstrated for the first time that the inhibition of FADS1 leads to a reduced conversion of DGLA to AA and induces an ER stress response. Reciprocally, extended ER stress also increases the transcription of FADS1, while inhibition of FADS1 reduces cells tolerance to ER stress inducers, leading to cell death. These data suggest that FADS1 expression is required to rescue RCC cells from persistent ER stress. This is noteworthy, because kidney cancers are known to be sensitive to hypoxia signals as well as anti-angiogenic agents, suggesting a potentially high basal level of ER stress in these cancers. As mentioned above, our previous analysis has shown an increased FADS1 expression in kidney cancers with a pattern of expression level in normal tissues<primary tumors<metastatic tumors<recurrent tumors [7]. Combined with the observations from our current study, these data may reflect an escalating dependence of FADS1 expression in response to an increased ER stress level during the progression of kidney cancers. Previously we also demonstrated that FADS1 expression is significantly associated with poor patient survival among all three independent cohorts of kidney cancer patients in TCGA [7]. Our studies hence collectively identify FADS1 as a key gene supporting the growth of renal cancer cells and as a therapeutic target for cancer treatment.

Within the ER membrane, three primary pathways are known to become active during ER stress: PERK, ATF6, and IRE1 [66]. Activation of these sensors sends signals to the nucleus, ultimately modifying cellular function and growth. Our data suggest that FADS1 inhibition predominantly activates the ATF4-ATF3 axis, which is under the PERK-mediated UPR pathway, possibly culminating in increased ATF3 expression within the nucleus. ATF3, being a key transcription factor, exerts diverse roles in cellular metabolism and function, including regulation of glucose metabolism, lipogenesis, immune response, and modulation of ER stress-induced responses [67,68]. In the context of cancer cells, ATF3 was demonstrated to have differential roles across various cancer types. For example, ATF3 functions as an oncogene in prostate cancer, where its high expression is associated with increased cell proliferation in response to androgen stimulation [68,69]. In breast cancer, elevated ATF3 expression induces the expression of MMP13, TWIST, Slug, and Snail, thereby modulating tumor metastasis [68,70]. In contrast, ATF3 acts as a tumor suppressor in lung cancer by inducing cancer cell apoptosis via activation of DR5 [68,71,72]. Similar to lung cancer, ATF3 functions as a tumor suppressor in colon and liver cancer by limiting cell proliferation and inducing apoptosis [68,73,74]. Our study supports ATF3 as a tumor suppressor in RCC that restrains renal cancer cell proliferation. How ATF3 regulates cell cycle and cell proliferation by mediating the effect of FADS1 inhibition remains to be further investigated. Interestingly, inhibiting SCD1 to block MUFA production was also shown to induce ER stress [75,76], in which ATF3 plays a critical role in mediating the downstream cellular responses in both cancer and other health conditions [9,65,77–79]. Therefore, the findings in our present study suggest that LC-PUFA production regulated by FADS1, plays a similar role to that of MUFA production controlled by SCD1. Collectively, our study indicates that fatty acids desaturation for either MUFA or PUFA production is a fundamental mechanism for maintaining ER homeostasis and function.

It should be noted that fatty acids may also directly contribute to cell proliferation and regulation of cell metabolism. In stark contrast to normal cells, cancer cells exhibit an insatiable demand for energy, membrane constituents, and regulatory factors to facilitate their rapid proliferation [80–82]. Consequently, extensive metabolic reprogramming becomes imperative to sustain this aberrant growth. A plethora of studies have underscored the critical involvement of fatty acid metabolism, encompassing both biosynthesis and desaturation of fatty acids, in fueling cancer cell proliferation [83–86]. More importantly, LC-PUFAs and their lipid derivatives e.g. eicosanoids, prostaglandins, and leukotrienes, have been broadly demonstrated to directly regulate cancer cell proliferation and cell cycle [1,87]. Our study demonstrates that FADS1 inhibition or knockdown leads to reduced production of AA metabolites, which possibly further reduces the cellular levels of downstream AA derivatives. This potential lipidomic remodeling and its role in regulating cell proliferation and cell cycle should be further explored in future studies.

We also noticed that FADS1 inhibition induces cell cycle arrest instead of cell death. This may be due to RCC cells, in culture, having a lower intrinsic baseline ER stress as compared to an RCC tumor *in vivo*. Therefore, cultured RCC cells might be less dependent on FADS1 expression. Indeed, when pre-treating the cells with ER stress inducers, FADS1 expression is increased, and FADS1 inhibition at this point induces cell apoptosis, as indicated by the increased cCASP3^+^ cells. Also, some studies have demonstrated that FADS1 inhibition or reduced expression enhances cancer cell sensitivity [88]. *In vivo*, due to the harsh microenvironment of kidney cancers, it is highly likely that cancer cells in the tumor possess an elevated ER stress level, thus an escalated dependence on FADS1 function, as indicated by the aforementioned increased FADS1 expression in more advanced stages of RCC [7]. Therefore, RCC tumors should be sensitive to FADS1 inhibition. Studies are ongoing in our lab to explore the potential impact of hypoxia, nutrient deprivation and existing anti-RCC drugs on ER stress in RCC cells, as well as the sensitivity of cells under these conditions to FADS1 inhibition.

To further investigate metabolic alterations mediating the impact of FADS1 inhibition on cell cycle arrest and ER stress, we conducted a metabolomic analysis in control and FADS1-KD 786-o cells. We observed a significant reduction in the levels of nucleotides and their related intermediate molecules in FADS1-KD cells. Previous studies have demonstrated that the availability of nucleotides is a critical factor in cell cycle progression [89–91]. Therefore, the reduced nucleotides biosynthesis may restrain DNA synthesis, which explains the cell cycle arrest to G1-S phase following FADS1 inhibition. However, why FADS1-KD results in depletion of nucleotides requires further investigation. Since our targeted metabolomics platforms only focus on a limited number of metabolites, especially those involved in energy metabolism, our results do not exclude the possibility that FADS1 may have impact on metabolic shift of cells that is independent of its influence on ER stress. However, our study indeed demonstrates that FADS1-KD cells exhibit a decrease in the level of UDP-GlcNAc. This sugar nucleotide is the product of the hexosamine biosynthetic pathway (HBP) and is the key substrate of protein N-glycosylation in the ER lumen [92]. Protein N-glycosylation is known to regulate protein folding and a diminished intracellular UDP-GlcNAc level may trigger the UPR signaling and induces ER stress [93]. This finding underscores the intricate connections between FADS1 function, metabolic remodeling, and cell stress. Future experiments are needed to explore how FADS1 and ATF3 lead to the metabolic remodeling for these pathways.

There are a few limitations in our study that require further investigation. For example, the cell cycle arrest and response pattern to ER stress between the 780-o and A498 cells are slightly different, which may reflect the variable susceptibility to these phenotypic responses between the two cells. Logically, different individuals or cells may possess different sensitivity to ER stress or cell cycle control. Notably, we previously demonstrated that key mutations e.g. TP53 in tumors are significantly correlated with FADS1 expression [7]. And it is known that 786-o but not A498 cell carries P53 mutations. Cellular sensitivity to FADS1 inhibitors may also contribute to the differences between the two cells as demonstrated in Supplementary Figure 1. Also, the ATF3 levels in the xenograft tumors developed from the FADS1-KD cells are not significantly different from that of the control tumors, though an apparent trend can be observed (5 out of 7 FADS1-KD tumors demonstrated an elevated level of ATF3), which may be largely due to the potential sex-specific differences, tumor-specific extent of ER stress, and the small sample size. In addition, while the animal study indeed provides proof-of-concept evidence, the study is relatively short and does not exclude the possibilities that ER stress-independent factors, e.g. metabolic activity or immune responses, influence the tumor growth. A long-term pharmacological treatment model *in vivo* may allow to better evaluate the therapeutic value of FADS1-targeting treatment. And using mouse tumor cells in immunocompetent mice may further help to assess the microenvironmental and immunological changes during FADS1 inhibition. These studies are now ongoing in the lab.

In summary, our findings demonstrate that FADS1 inhibition effectively curtails renal cancer cell proliferation *in vitro* as well as tumor formation *in vivo*, primarily through the activation of the PERK-ATF4-ATF3 axis-mediated ER stress. Our study highlights FADS1-ER stress as a key therapeutic target for renal cancer, paving the way for novel drug discovery and development.

## Supplementary Material

1

## Data Availability

Data will be made available on request.
