# Supplementary material for: Targeting polyunsaturated fatty acids desaturase FADS1 inhibits renal cancer growth via ATF3-mediated ER stress response
Source: Biomed Pharmacother. Author manuscript; Available in PMC 2025 May 1. (PMC12034426; doi:10.1016/j.biopha.2025.118006)
Supplement: 1 [file NIHMS2076393-supplement-1.docx]

**
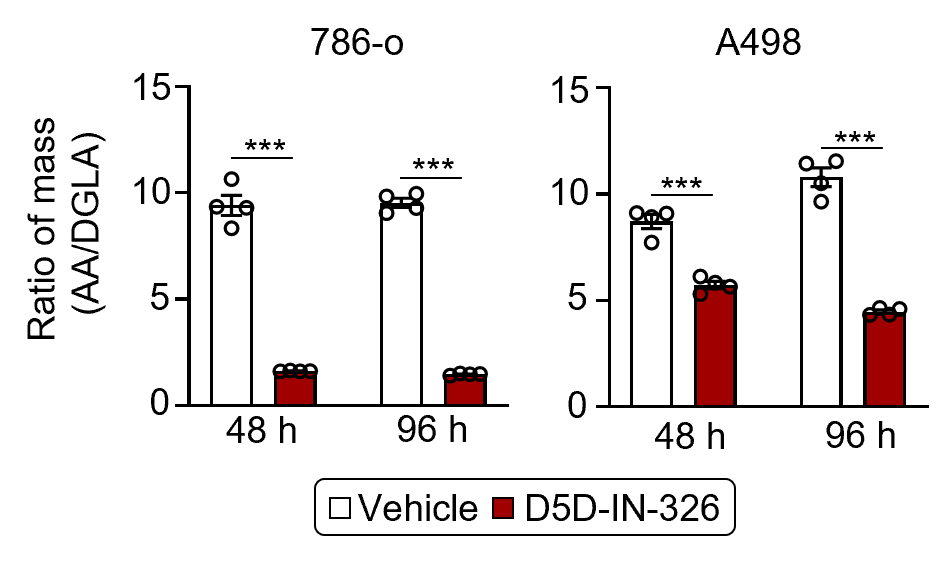
**

**Supplementary Figure 1. D5D-IN-326 inhibits biosynthesis of intracellular LC-PUFAs.**

The column graph showing the ratio of AA/DGLA in 786-o and A498 cells treated with 2,000 nM D5D-IN-326 treatment (vehicle as control) for 48 hours and 96 hours (mean ± standard error). Statistical analysis was performed using two-tailed unpaired Student’s t test. ***P<0.001.


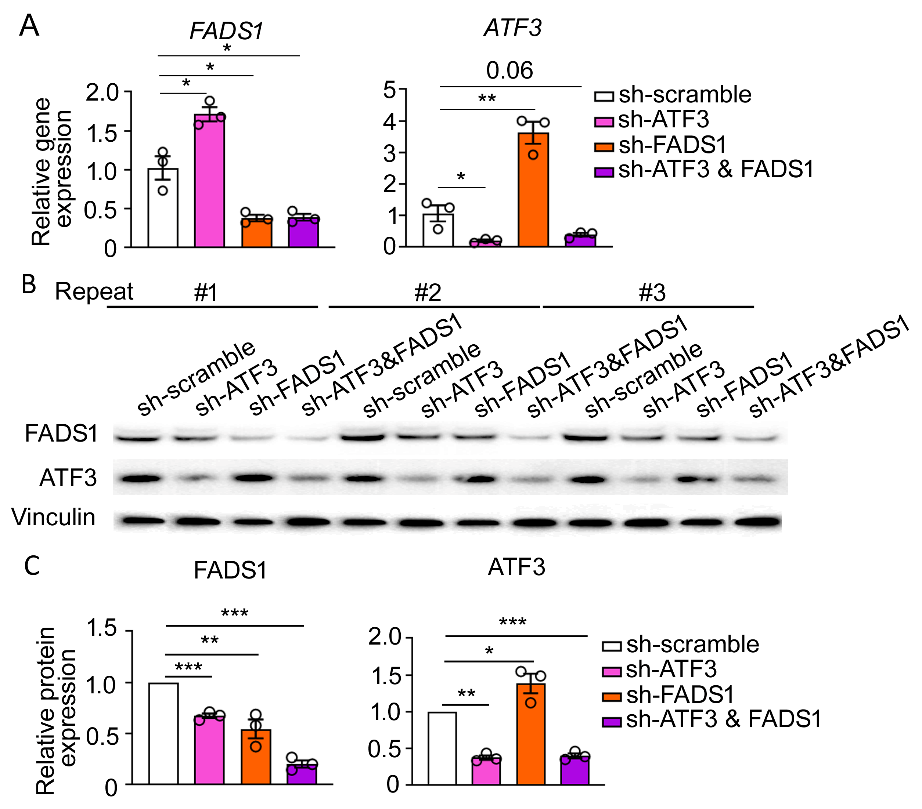


**Supplementary Figure 2. Evaluation of the sh-ATF3, sh-FADS1, and sh-ATF3 + sh-FADS1 786-o cells**

(A) The column graph showing relative ATF3 and FADS1 gene expression in sh-scramble, sh-ATF3, sh-FADS1, sh-ATF3 & FADS1 786-o cells. Data were normalized to the sh-scramble group. Statistical analysis was conducted using two-tailed unpaired Student’s t test. *P<0.05; **P<0.01. (B) Western blot images illustrating expression of ATF3 and FADS1 protein (Vinculin as the housekeeping protein) in sh-scramble, sh-ATF3, sh-FADS1, sh-ATF3 + sh-FADS1 786-o cells. (C) The column bar graphs showing the relative quantification of ATF3 and FADS1 proteins in sh-scramble, sh-ATF3, sh-FADS1, sh-ATF3 + sh-FADS1 786-o cells based on Western blot analysis (mean ± standard error). Each ATF3 and FADS1 protein level was normalized to its corresponding sh-scramble group in each experimental replicate. Consequently, all sh-scramble values were set to '1,' and therefore no error bar is presented from the control groups. Statistical analysis conducted using two-tailed unpaired Student’s t test. *P<0.05; **P<0.01; ***P<0.001.


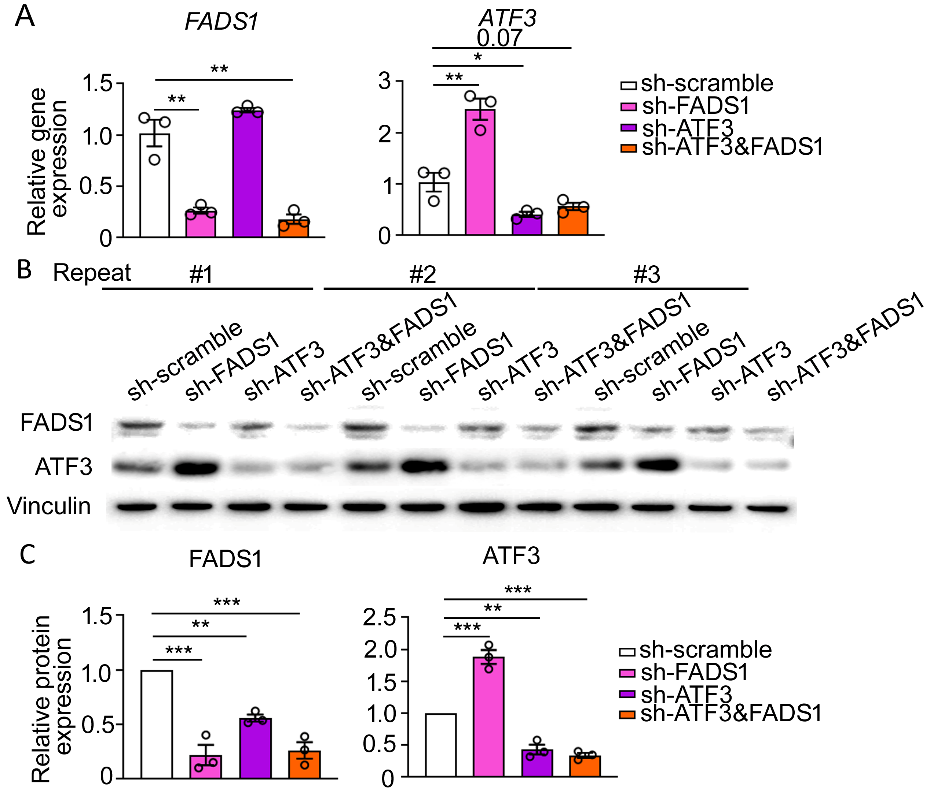


**Supplementary Figure 3. Evaluation of the sh-ATF3, sh-FADS1, and sh-ATF3 + sh-FADS1 A498 cells**

(A) The column graph showing relative *ATF3* and *FADS1* gene expression in sh-scramble, sh-ATF3, sh-FADS1, sh-ATF3 & FADS1 A498 cells. Data were normalized to the sh-scramble group. Statistical analysis was conducted using two-tailed unpaired Student’s t test. *P<0.05; **P<0.01. (B) Western blot images illustrating expression of ATF3 and FADS1 protein (Vinculin as the housekeeping protein) in sh-scramble, sh-ATF3, sh-FADS1, sh-ATF3 + sh-FADS1 A498 cells. (C) The column bar graphs showing the relative quantification of ATF3 and FADS1 proteins in sh-scramble, sh-ATF3, sh-FADS1, sh-ATF3 + sh-FADS1 A498 cells based on Western blot analysis (mean ± standard error). Each ATF3 and FADS1 protein level was normalized to its corresponding sh-scramble group in each experimental replicate. Consequently, all sh-scramble values were set to '1,' with no error bar for the control group. Statistical analysis conducted using two-tailed unpaired Student’s t test. *P<0.05; **P<0.01; ***P<0.001.


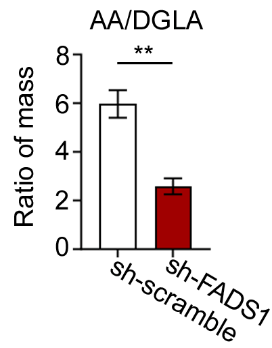


**Supplementary Figure 4. FADS1-knockdown inhibits biosynthesis of intracellular LC-PUFAs**

The column graph showing the ratio of AA/DGLA in sh-scramble and sh-ATF3 786-o cells (mean ± standard error). Statistical analysis performed using two-tailed unpaired Student’s t test. **P<0.01.


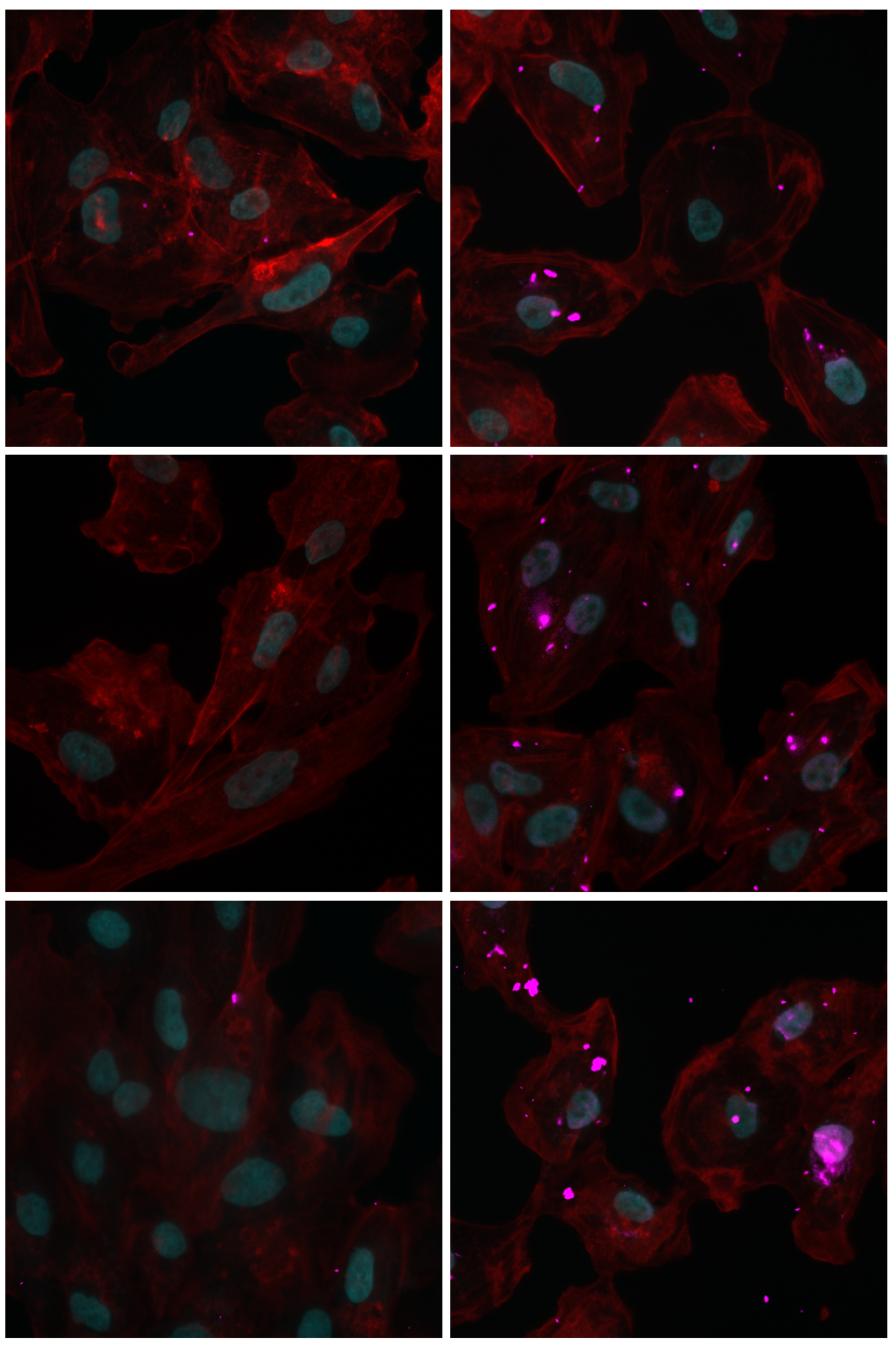


Vehicle

p-ATF4

A498

D5D-IN-326

p-eIF2α

p-PERK

Nuclei

Phalloidin

**Supplementary Figure 5. Inhibition of FADS1 activities the A498 ER stress.**

The representative fluorescence images showing the expression of the p-PERK, p-eIF2α, and p-ATF4 staining in the A498 cells treated with 2,000 nM D5D-IN-326 (vehicle as control) for 72 hours (cells were labelled with DAPI and phalloidin). Scar bar: 5 μm.


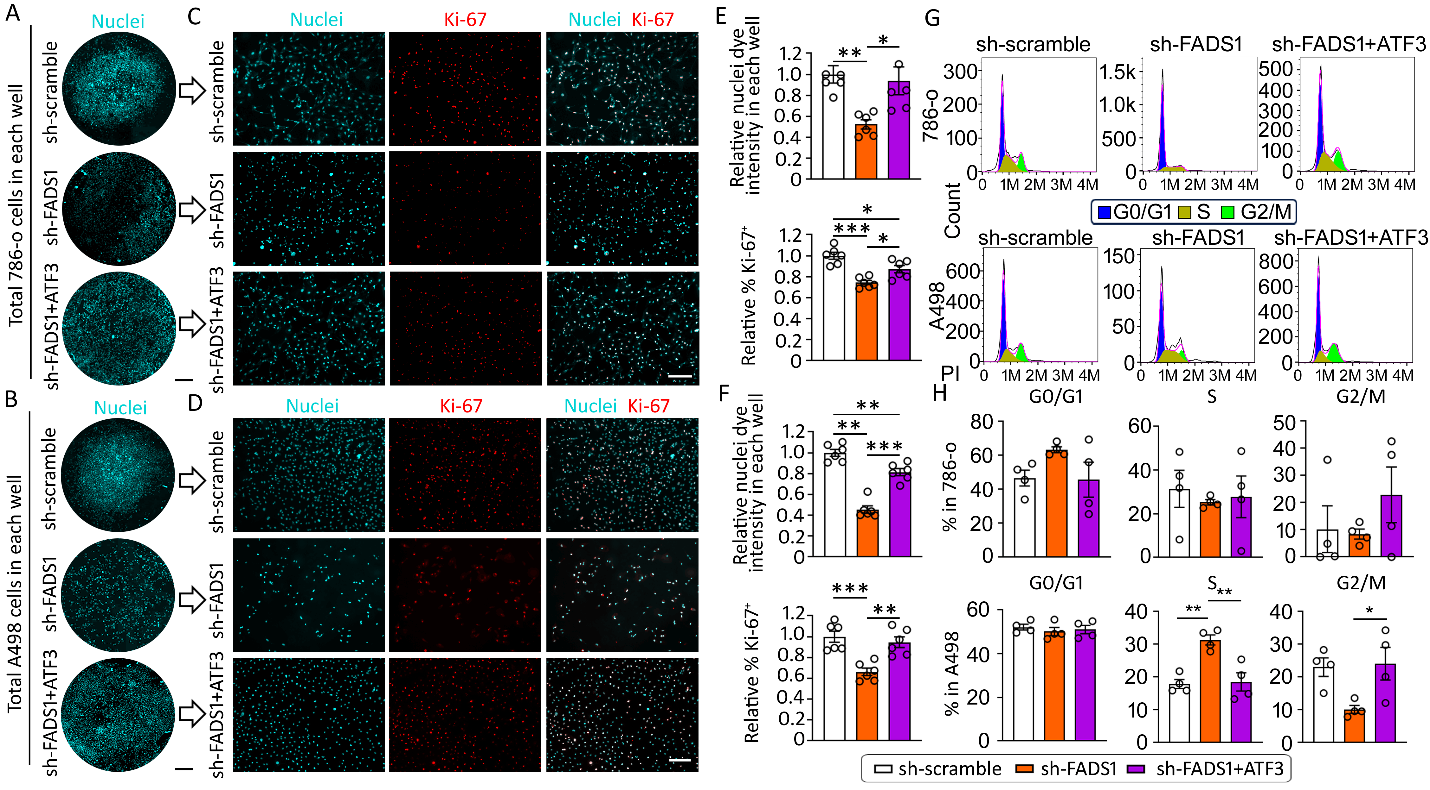


**Supplementary Figure 6. Co-knockdown of *ATF3* and *FADS1* rescues reduced cell proliferation induced by FADS1 inhibition.**

(A) Representative overview images of nuclei staining showing scramble control (sh-scramble), *Fads1* knockdown (sh-FADS1), and ATF3 & FADS1 co-knockdown (sh-ATF3 + sh-FADS1) of 786-o cells or (B) A498 cells in each well. (C) Representative immunofluorescence images illustrating expression of Ki-67 in cells treated with sh-scramble, sh-FADS1, and sh-ATF3 + sh-FADS1 786-o cells or (D) A498 cells. Scale bar: 10 mm in (A) and 100 μm in (B). (E) The column bar graphs showing the relative quantification of whole nuclei staining intensity and percentage of Ki-67 positive cells in sh-scramble, sh-FADS1, and sh-ATF3 + sh-FADS1 786-o cells or (F) A498 cells (mean ± standard error). Data were normalized to the sh-scramble group. Statistical analysis was conducted using Tukey’s multiple comparisons test. *P<0.05; **P<0.01; ***P<0.001. (G) Representative histogram showing the expression of the propidium iodide (PI) in sh-scramble, sh-FADS1, and sh-ATF3 + sh-FADS1 786-o (top) or A498 (bottom) cells. The different stages of cell cycle (G0/G1, S, G2/M stage) were determined by the expression of PI, as shown in the plot. (H) The column bar graphs showing the percentage of each cell cycle stage (G0/G1, S, and G2/M) in sh-scramble, sh-FADS1, and sh-ATF3 + sh-FADS1 786-o (top) or A498 (bottom) cells (mean ± standard error). Statistical analysis conducted using Tukey’s multiple comparisons test. *P<0.05; **P<0.01.


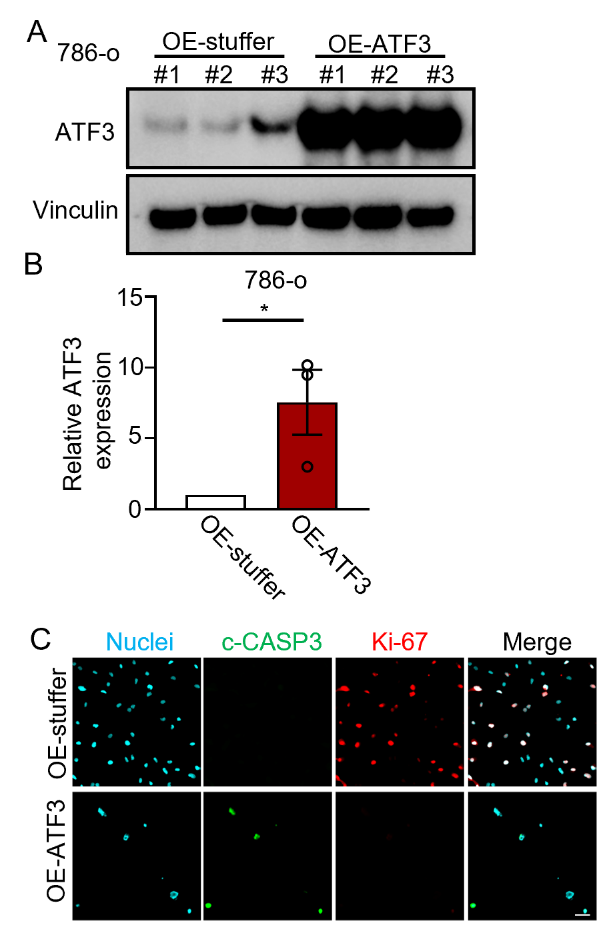


**Supplementary Figure 7. Substantial ATF3 overexpression leads to rapid cell death**

(A) Western blot images illustrating substantial expression of ATF3 (Vinculin as the housekeeping protein) in OE-stuffer and OE-ATF3 786-o cells. (B) The column bar graphs showing the relative quantification of ATF3 proteins in OE-stuffer and OE-ATF3 786-o cells based on Western blot analysis (mean ± standard error). Each ATF3 and FADS1 protein level was normalized to its corresponding OE-stuffer group in each experimental replicate. Consequently, all OE-stuffer values were set to '1,' with no error bar for this group. Statistical analysis conducted using two-tailed unpaired Student’s t test. *P<0.05. (C) Representative immunofluorescence images illustrating expression of c-CASP3 and Ki-67 in OE-stuffer and OE-ATF3 786-o cells. Scale bar: 10 μm.

**
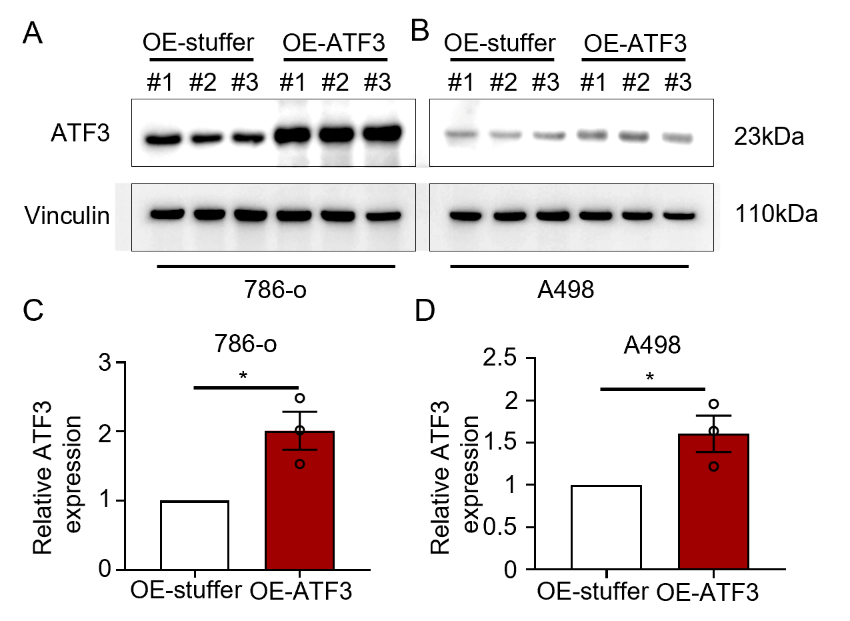
**

**Supplementary Figure 8. Evaluation of the OE-stuffer and OE-ATF3 786-o cells**

(A) Western blot images illustrating expression of ATF3 (Vinculin as the housekeeping protein) in OE-stuffer and OE-ATF3 786-o and A498 (B) cells. (C) The column bar graphs showing the relative quantification of ATF3 proteins in OE-stuffer and OE-ATF3 786-o and A498. Each ATF3 and FADS1 protein level was normalized to its corresponding OE-stuffer group in each experimental repeat. Consequently, all OE-stuffer values were set to '1,' with no error bar for the this group. (D) cells based on Western blot analysis (mean ± standard error). Data was normalized to the OE-stuffer group. Statistical analysis conducted using two-tailed unpaired Student’s t test. *P<0.05; **P<0.01; ***P<0.001.

**
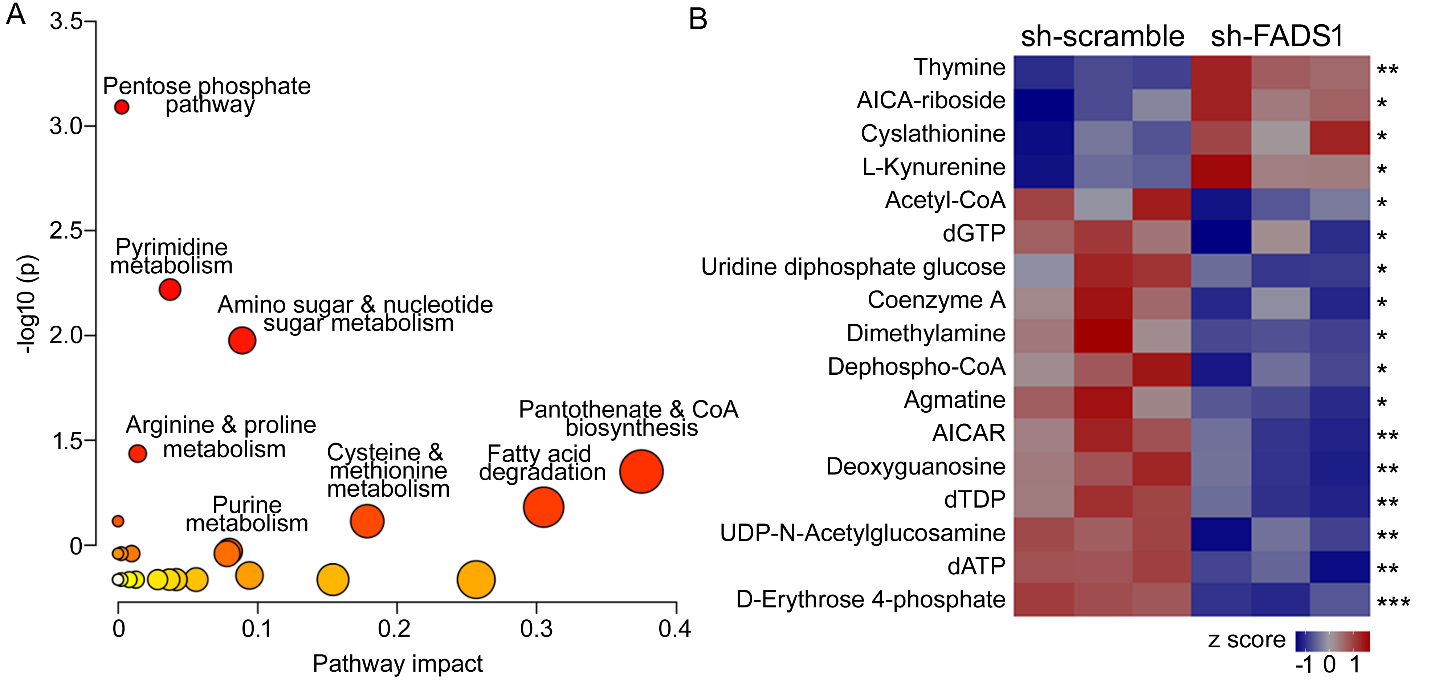
**

**Supplementary Figure 9: Impact of FADS1 knockdown on metabolomic profile of 786-o cells.**

(A) Metabolomic pathway analysis plot revealing enriched (p<0.05) metabolic pathways in 786-o cells with FADS1 knockdown. (B) Heatmap plot illustrating the metabolites with significant change in their levels in between sh-scramble and sh-FADS1 786-o cells (relative to z score). Statistical analysis was conducted using a two-tailed unpaired Student’s t-test. *P<0.05, **P<0.01, ***P<0.001.


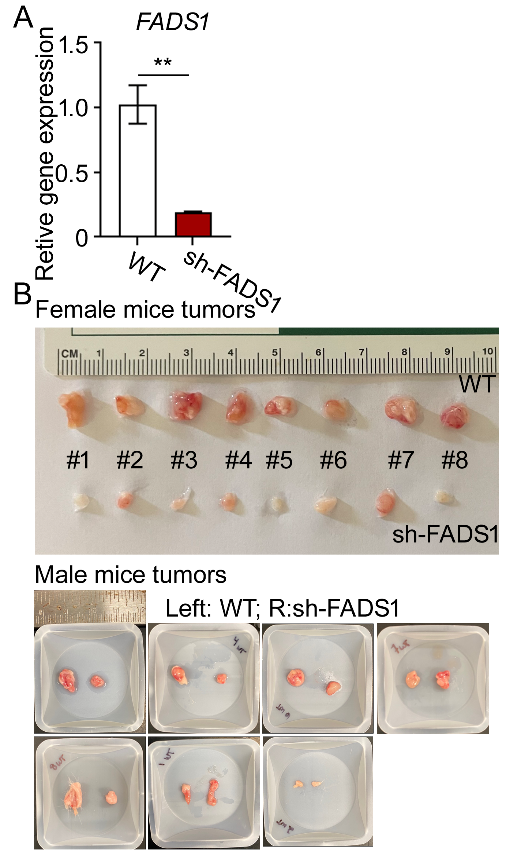


**Supplementary Figure 10: Images of extracted tumors from male and female mice**

(A) The column graph showing the *FADS1* gene expression in WT and sh-FADS1 786-o cells (mean ± standard error; *in vivo* study only). Data were normalized to the WT group. Statistical analysis performed using two-tailed unpaired Student’s t test. **P<0.01. (B) The images of extracted subcutaneous tumors from (top) female (8 pairs of tumors; upper tumors were 786-o WT and lower tumors were 786-o sh-FADS1) and (bottom) male mice (7 pairs of tumors; one mouse did not develop any tumor; left tumors were 786-o WT and right tumors were 786-o sh-FADS1).

**Supplemental Table 1. Sequences for the shRNA experiments (All sequences are provided in 5’ to 3’ orientation)**

| **Name** | **Target** | **Hairpin sequence** |
| --- | --- | --- |
| sh-ATF3  (SCBT; Cata# sc-29757-V) | *ATF3* | GATCCCGACGAGAAAGAAATAAGATTCAAGAGATCTTATTTCTTTCTCGTCGTTTTT |
|  |  | GATCCGTGTGAATGCTGAACTGAATTCAAGAGATTCAGTTCAGCATTCACACTTTTT |
|  |  | GATCCCTCTCCACTCAATGTCTTATTCAAGAGATAAGACATTGAGTGGAGAGTTTTT |
| sh-FADS1  (SCBT; Cata# sc-96474-V) | *FADS1* | GATCCGGATAGGTATGACCTATGTTTCAAGAGAACATAGGTCATACCTATCCTTTTT |
|  |  | GATCCCTCTAGGCATATTGATCATTTCAAGAGAATGATCAATATGCCTAGAGTTTTT |
|  |  | GATCCCCATGGAGAGGTTTGTCAATTCAAGAGATTGACAAACCTCTCCATGGTTTTT |
| sh-FADS1  (*in vivo*) | *FADS1* | GATCCCGCCTTGTGAAGAAGTATATGAATTCAAGAGA TTCATATACTTCTTCACAAGGTTTTT |
| OE-stuffer (VectorBuilder) | Control | GTCGTTTTACAACGTCGTGACTGGGAAAACCCTGGCGTTACCCAACTTAATCGCCTTGCAGCACATCCCCCTTTCGCCAGCTGGCGTAATAGCGAAGAGGCCCGCACCGATCGCCCTTCCCAACAGTTGCGCAGCCTGAACGGCGAGTGGCGCTTTGCCTGGTTTCCGGCACCAGAAGCGGTGCCGGAAAGCTGGCTGGAGTGCGATCTTCCTGAGGCCGATACTGTCGTCGTCCCCTCAAACTGGCAG |
| OE-ATF3 (VectorBuilder) | *ATF3* | ATGATGCTTCAACACCCAGGCCAGGTCTCTGCCTCGGAAGTGAGTGCTTCTGCCATCGTCCCCTGCCTGTCCCCTCCTGGGTCACTGGTGTTTGAGGATTTTGCTAACCTGACGCCCTTTGTCAAGGAAGAGCTGAGGTTTGCCATCCAGAACAAGCACCTCTGCCACCGGATGTCCTCTGCGCTGGAATCAGTCACTGTCAGCGACAGACCCCTCGGGGTGTCCATCACAAAAGCCGAGGTAGCCCCTGAAGAAGATGAAAGGAAAAAGAGGCGACGAGAAAGAAATAAGATTGCAGCTGCAAAGTGCCGAAACAAGAAGAAGGAGAAGACGGAGTGCCTGCAGAAAGAGTCGGAGAAGCTGGAAAGTGTGAATGCTGAACTGAAGGCTCAGATTGAGGAGCTCAAGAACGAGAAGCAGCATTTGATATACATGCTCAACCTTCATCGGCCCACGTGTATTGTCCGGGCTCAGAATGGGAGGACTCCAGAAGATGAGAGAAACCTCTTTATCCAACAGATAAAAGAAGGAACATTGCAGAGCTAA |

**Supplemental Table 2. Primers for the RT-qPCR analyses**

| **Gene** | **NCBI ID** | **Forward primer** | **Reverse primer** |
| --- | --- | --- | --- |
| *FADS1* | 3992 | CCTGGAAAGCAACTGGTTTGTG | GAAGGCAGACTTGTGGACATTG |
| *PPIA* | 5478 | AGGTCCCAAAGACAGCAGAA | GAAGTCACCACCCTGACACA |
| *ATF3* | 467 | CCTCTGCGCTGGAATCAGTC | TTCTTTCTCGTCGCCTCTTTTT |
| *ATF4* | 468 | CCCTTCACCTTCTTACAACCTC | TGCCCAGCTCTAAACTAAAGGA |

**Supplemental Table 3. Primary antibodies for immunofluorescence assays**

| **Antibody** | **Species** | **Dilution** | **Company (Catalog #)** |
| --- | --- | --- | --- |
| ATF3 | Mouse | 1:100 | Santa Cruz Biotechnology (sc-518032) |
| CASP3 | Rabbit | 1:200 | Abcam (ab32351) |
| FADS1 | Rabbit | 1:200 | Abcam (ab126706) |
| Ki-67 | Rat | 1:100 | Thermofisher (14-5698-82) |
| p-PERK | Rabbit | 1:200 | Thermofisher (PA5-40294) |
| p-eIF2A | Rat | 1:200 | R&D (MAB39971) |
| p-ATF4 | Rabbit | 1:200 | Thermofisher (PA5-36624) |

**Supplemental Table 4. Secondary antibodies for immunofluorescence assays**

| **Antibody** | **Conjugate** | **Dilution** | **Company (Catalog #)** |
| --- | --- | --- | --- |
| Donkey anti mouse | Alexa 647 | 1:500 | Jackson ImmunoResearch (715-606-150) |
| Donkey anti rabbit | Alexa 488 | 1:500 | Jackson ImmunoResearch (711-546-152) |
| Donkey anti rat | Cyanine Cy3 | 1:500 | Jackson ImmunoResearch (712-166-150) |

**Supplemental Table 5. Primary antibodies for western blot assay**

| **Antibody** | **Species** | **Dilution** | **Company (Catalog #)** |
| --- | --- | --- | --- |
| ATF3 | Rabbit | 1:500 | Abcam (ab254268) |
| ATF4 | Rabbit | 1:1000 | Abcam (ab270980) |
| ATF6 | Mouse | 1:1000 | Novus biologicals (NBP1-40256) |
| BIP | Rabbit | 1:1000 | Novus biologicals (NBP1-06277) |
| CHOP | Rabbit | 1:1000 | Novus biologicals (NBP2-13172) |
| FADS1 | Rabbit | 1:1000 | Abcam (ab126706) |
| GAPDH | Rabbit | 1:1000 | Cell signaling (2118s) |
| pIRE1α | Rabbit | 1:1000 | Novus biologicals (NB100-2323) |
| XBP1 | Rabbit | 1:1000 | Novus biologicals (NBP1-77681) |
| Vinculin | Rabbit | 1:1000 | Cell signaling (4650S) |

**Supplemental Table 6. Secondary antibodies for western blot assays**

| **Antibody** | **Dilution** | **Company (Catalog #)** |
| --- | --- | --- |
| Anti-mouse, HRP linked | 1:1000 | Cell signaling (7076s) |
| Anti-rabbit, HRP linked | 1:1000 | Cell signaling (7074s) |
